# Novel Spectroscopic and Electrochemical Sensors and Nanoprobes for the Characterization of Food and Biological Antioxidants

**DOI:** 10.3390/s18010186

**Published:** 2018-01-11

**Authors:** Reşat Apak, Sema Demirci Çekiç, Ayşem Üzer, Saliha Esin Çelik, Mustafa Bener, Burcu Bekdeşer, Ziya Can, Şener Sağlam, Ayşe Nur Önem, Erol Erçağ

**Affiliations:** 1 Department of Chemistry, Faculty of Engineering, Istanbul University, Avcilar, 34320 Istanbul, Turkey; sema@istanbul.edu.tr (S.D.Ç.); auzer@istanbul.edu.tr (A.Ü.); secelik@istanbul.edu.tr (S.E.Ç.); mbener@istanbul.edu.tr (M.B.); burcubek@istanbul.edu.tr (B.B.); ziya.can@istanbul.edu.tr (Z.C.); sener.saglam@istanbul.edu.tr (Ş.S.); ayse.tufan@istanbul.edu.tr (A.N.Ö.); 2 Turkish Academy of Sciences (TUBA), Piyade Sok., No. 27, Cankaya, 06550 Ankara, Turkey; 3 Aytar Cad., Fecri Ebcioglu Sok., No. 6/8, Levent, 34340 Istanbul, Turkey; e.ercag58@gmail.com

**Keywords:** nanoprobes, antioxidant capacity, oxidative status, CUPRAC antioxidant assay, DMPD oxidant assay, colorimetric and electrochemical sensors

## Abstract

Since an unbalanced excess of reactive oxygen/nitrogen species (ROS/RNS) causes various diseases, determination of antioxidants that can counter oxidative stress is important in food and biological analyses. Optical/electrochemical nanosensors have attracted attention in antioxidant activity (AOA) assessment because of their increased sensitivity and selectivity. Optical sensors offer advantages such as low cost, flexibility, remote control, speed, miniaturization and on-site/in situ analysis. Electrochemical sensors using noble metal nanoparticles on modified electrodes better catalyze bioelectrochemical reactions. We summarize the design principles of colorimetric sensors and nanoprobes for food antioxidants (including electron-transfer based and ROS/RNS scavenging assays) and important milestones contributed by our laboratory. We present novel sensors and nanoprobes together with their mechanisms and analytical performances. Our colorimetric sensors for AOA measurement made use of cupric-neocuproine and ferric-phenanthroline complexes immobilized on a Nafion membrane. We recently designed an optical oxidant/antioxidant sensor using *N*,*N*-dimethyl-*p*-phenylene diamine (DMPD) as probe, from which ROS produced colored DMPD-quinone cationic radicals electrostatically retained on a Nafion membrane. The attenuation of initial color by antioxidants enabled indirect AOA estimation. The surface plasmon resonance absorption of silver nanoparticles as a result of enlargement of citrate-reduced seed particles by antioxidant addition enabled a linear response of AOA. We determined biothiols with Ellman reagent−derivatized gold nanoparticles.

## 1. Design Strategies and Accomplishments for Colorimetric Sensors and Nanoprobes of Antioxidant Detection

The accumulation of reactive oxygen/nitrogen species (ROS/RNS) in the organism, unless counterbalanced by intrinsic and dietary antioxidants, may cause oxidative damage to biological macromolecules and cellular membranes under ‘oxidative stress’ conditions, eventually giving rise to certain human diseases, especially cardiovascular and neurodegenerative diseases and some types of cancer. Therefore, measuring antioxidant capacity/activity of food extracts and biological fluids is important for human health. Various antioxidant assay methods, extensively spectroscopic techniques, applied to aqueous solutions have been developed to determine total antioxidant capacity (TAC) or antioxidant activity in a wide range of matrices such as biological fluids, food, and plant extracts. These methods may be broadly classified as electron transfer (ET)-based assays and hydrogen atom transfer (HAT)-based assays, without distinct boundaries. ET-assays (e.g., CUPRAC, FRAP, ferricyanide) and ET-HAT mixed-mode assays (e.g., DPPH and ABTS) usually exploit the formation or fading of colored species. Tests measuring the scavenging ability of antioxidants toward ROS/RNS cover both mechanisms as well as other side reactions targeting at initiators of reactive species, therefore those assays focusing on the measurement of reactive species as well as on their scavenger antioxidants form a special category. In this review, colorimetric sensors and nanoprobes were essentially classified according to the reaction mechanisms mentioned above, naturally with a tolerance for unavoidable interwining of these mechanisms. 

### 1.1. Electron Transfer-Based Colorimetric Sensors and Nanoprobes for Total Antioxidant Capacity Assay

#### 1.1.1. Optical Sensors

These sensors attract attention nowadays because they offer a number of advantages in many application processes [1]. Compared with classical instrumental methods, optical sensors have many advantages such as low cost, flexibility, speed, remote control, miniaturization and on site/in situ analysis. Optical sensors evaluate analytical information by using optical transduction techniques (absorbance, reflectance, luminescence, etc.) and may be suitable for colored and turbid samples. Reagent-based sensors (optrodes) are suitable for simple, rapid and low-cost analysis with high sensitivity and selectivity, and can also be convertible to the form of test kits and easy to use test strips for practical assessment [2,3]. However, aqueous-solution based spectrophotometric methods may require larger reagent volumes and may not work in turbid solutions, and therefore, colored complex formation on the surface of a solid sensor may be advantageous. 

**Figure 1:**
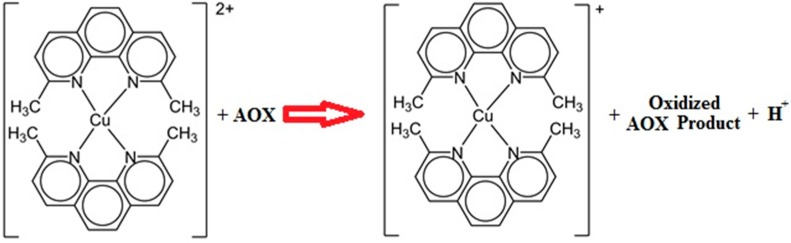
The CUPRAC reaction of Cu(II)-neocuproine complex with antioxidants, producing the yellow-orange colored Cu(I)-neocuproine chelate (λ_max_ = 450 nm).

Our research group introduced the cupric ion reducing antioxidant capacity (CUPRAC) assay in 2004 [4], and the CUPRAC method is currently being widely used in leading world research centers of food science and antioxidant research. CUPRAC is an ET-based method, utilizing the Cu(II)-Cu(I) reduction in the presence of antioxidants, where cupric-neocuproine is converted into the highly absorbing (yellow-orange colored) cuprous-neocuproine chelate by receiving an electron from antioxidant polyphenols, thiols, or vitamins (Figure 1).

**Figure 2:**
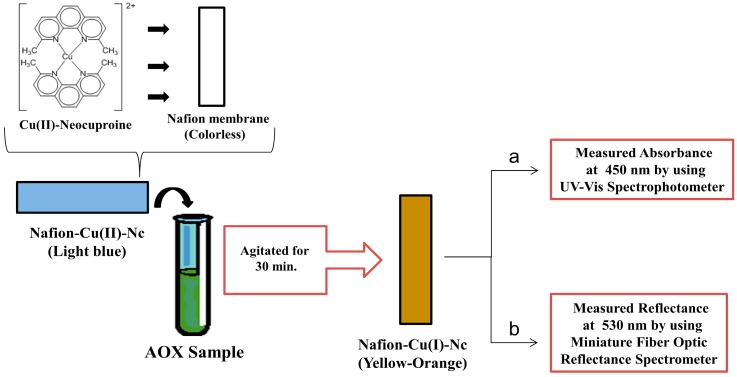
Schematic diagram of CUPRAC antioxidant sensors: the measurement of the analytical signal (**a**) for absorptimetric sensor and (**b**) for reflectometric sensor.

We later developed a low-cost optical antioxidant sensor (CUPRAC sensor) by immobilizing the copper(II)-neocuproine (Cu(II)-Nc) reagent onto a perfluorosulfonate cation-exchange polymer membrane matrix (Nafion), and the colored Cu(I)-Nc cation was produced on the membrane without diffusing into solution [5]. The Cu(II)→Cu(I) reduction by electron-donating antioxidants in the presence of neocuproine ligand did not cause a drastic change in the coordination geometry of the copper-chelate, and therefore both cationic species were retained on the anionic membrane, and the color change took place on the sensor. This membrane sensor provided great ease and convenience to TAC determinations, like a sensitive pH-indicator paper strip (immersed in solution) on which a series of neutralization indicators were previously impregnated. The CUPRAC sensor utilizing the Cu(II)-Nc complex immobilized on the Nafion membrane could measure the TAC value both colorimetrically at 450 nm (Figure 2a) and reflectometrically at 530 nm (Figure 2b) [6]. As a distinct difference from absorptimetric sensor, the reflectometric sensor was applicable to non-transparent opaque media. 

Optical sensor based CUPRAC methods were validated through linearity, additivity, precision, and recovery, demonstrating that the assays are reliable and robust [5,6]. The precision, which is expressed as the relative standard deviation (RSD, %) in absorptimetric and reflectometric modes of measurement within the tested concentration range, were 6.5% and 5.0%, respectively. The recoveries of spiked antioxidants, namely quercetin, ascorbic acid and α-tocopherol, for the absorptimetric and reflectometric sensors varied in the ranges of 93−100% and 92−94%, respectively.

The limit of detection (LOD) values for TR by using absorptimetric and reflectometric CUPRAC sensors were found to be 1.01 and 0.53 μmol·L^−1^, respectively. The presence of 1000-fold citrate, oxalate, and tartarate ions that may be found in fruit juices and other food plant extracts did not interfere with the CUPRAC reaction forming the basis of sensor measurement.

Both of the CUPRAC sensors were used to screen the TAC values of some fruit juice samples and showed a promising potential for the preparation of antioxidant inventories of a wide range of food plants. The Trolox-equivalent antioxidant capacity (TEAC) coefficients of plant polyphenols were listed in Table 1.

In addition to food antioxidants, the sensor- and solution-based CUPRAC methods gave similar responses to plasma antioxidants such as α-tocopherol, uric acid and bilirubin. Of the three exceptional antioxidants yielding different TEAC values (Table 1), naringenin exhibited enhanced antioxidant power on Nafion possibly due to enhanced solubility in a non-polar environment, whereas catechin and rosmarinic acid showed significantly lower TEAC coefficients on the sensor membrane (than in solution). Catechin lacks the 2,3-double bond conjugated with the 4-oxo group responsible for electron delocalization, which is considered to be an important prerequisite for high antioxidant power. The superior antioxidant ability of quercetin results from the formation of a stable aryloxy radical, due to C2–C3 double bond and the resulting planar geometry which delocalizes the radical throughout the entire molecule, whereas A and B rings are perpendicular to each other in catechin. Considering the enforced planar geometry of copper(I)-neocuproine (which is actually a tetrahedral chelate, due to the sp^3^ hybridization of Cu(I) having d^10^ electron configuration) on the sensor membrane, lack of planarity of catechin is an important drawback playing part in the decreased antioxidant power of catechin. On the other hand, in spite of the four phenolic –OH groups and excellent conjugated structure of rosmarinic acid, its TEAC coefficient in the sensor assay decreased, probably due to its large molecular size being an important parameter in optical sensor response and its low pKa = 2.8, causing repellance from the negative perfluorosulfonate groups of Nafion. 

Another Nafion membrane-based TAC sensor recently developed in our laboratory utilized the Fe(III)-*o*-phenanthroline (Fe(III)-phen) reagent immobilized on Nafion, to which antioxidants donated an electron to convert it to the highly colored Fe(II)-phen complex, remaining on the membrane after electron-transfer [7]. The absorbance of the orange-red colored complex retained by Nafion was measured at 510 nm, yielding a high sensitivity for antioxidants (e.g., the LOD for trolox was 0.26 μmol·L^−1^) and good linearity over a wide concentration range. This sensor was especially useful for colored and turbid solutions like fruit juices, and was more sensitive than the solution-phase ferric-phenanthroline method because the membrane concentrated the colored species from a larger volume solution. The TEAC coefficients of the sensor-based Fe(III)-phen method were similar to those of the solution-based method, except for rosmarinic, ascorbic and uric acids, showing smaller TEAC values with the sensor, possibly related to the size and charge of the concerned antioxidants. The sensor gave additive responses to antioxidant mixtures, in accordance with Beer’s law. The proposed sensor is small and cheap, and easily convertible to kit format for in situ antioxidant screening of complex food matrices. 

Sensors capable of simultaneously measuring oxidative status and antioxidant capacity are rare in literature. Recently, we designed a solid membrane sensor for the simultaneous colorimetric determination of oxidative status and antioxidant activity, by immobilizing the pink-colored *N*,*N*-dimethyl-*p*-phenylenediamine (DMPD) quinonic cation radicals on a Nafion membrane [8]. Hydroxyl radicals generated in either a Fenton or UV/H_2_O_2_ system or superoxide anion radicals enzymatically generated by xanthine/xanthine oxidase reaction attacked the probe DMPD and oxidized it to DMPD-quinone (DMPD-Q^•+^), adsorbable on a cationic Nafion membrane. The mechanism of uptake of DMPD-Q^•+^ cationic species by the anionic R-SO_3_H^−^ groups of the sensor membrane was basically electrostatic retention, yielding a high distribution coefficient (i.e., meaning that all the colored species was held by the membrane without diffusing into solution). Antioxidants, when present, caused an absorbance decrease on the membrane due to their ROS scavenging action, giving rise to less DMPD-quinone production. The decrease in absorbance (ΔA) at λ_max_ = 514 nm of the membrane sensor in the presence of antioxidants enabled indirect TAC determination of complex samples, ΔA varying linearly with antioxidant concentration over a reasonable concentration range. Choosing three representative antioxidants in the high (epigallocatechin gallate), medium (quercetin) and low (*p*-coumaric acid) molar absorptivity range, the detection limit ranged within the concentration intervals of 0.2–14 μmol·L^−1^, depending on the radical scavenged. The method enabled simultaneous determination of oxidative status and antioxidant activity (as oxidants generated the colored DMPD-Q^•+^ and antioxidants attenuated this color formed on the membrane by scavenging ROS), and was validated in synthetic and real antioxidant mixtures.

Gavrilenko et al. [9] immobilized Fe(III)–1,10-phenanthroline (Fe(III)–*o*-phen) complex in a polymethacrylate matrix to manufacture a colorimetric TAC sensor, and the absorbance changes associated with the formation of orange-colored Fe(II)–*o*-phen chelate resulting from the reaction of antioxidants with the polymer-immobilized reagent was measured at 510 nm. Although the authors used this sensor for TAC quantitation, its sensitivity can be compared with that of the CUPRAC sensor (i.e., the molar absorptivities of ascorbic acid were ≈3450 and ≈17,100 L mol^−1^ cm^−1^ with respect to this specific Fe(III)–*o*-phen sensor and the CUPRAC sensor, respectively). This low sensitivity probably originated from partial inactivation of the redox-active ferric centre of the coordination complex during embedding in the polymer matrix. 

The use of chromogenic radicals immobilized on solid matrices for antioxidant activity assessment was described by Steinberg and Milardović [10]. DPPH^•^ (2,2-diphenyl-1-picrylhydrazyl) and galvinoxyl radical (GV^•^), as two stable chromogenic radicals, were immobilized in polymeric PVC (polyvinyl chloride) sensing film. The immobilized DPPH^•^ fades (i.e., changes color from purple to yellow) due to a mixed mechanism of electron/H-atom donation by polyphenols to the radical [11,12], resulting in a decrease of absorbance at 520 nm. The mentioned irreversible color change was traceable with the naked eye for DPPH^•^ but not for galvinoxyl radical (absorption maximum at 430 nm).

#### 1.1.2. Nano Sensors/Nanoprobes

Because of their small size (1–100 nm) and very high specific surface area, noble metal (Au, Ag, Pt, Pd, Rh, etc.) nanoparticles (NPs) have attracted much attention and found applications in diverse areas, including medicine, catalysis, textile engineering, biotechnology and bioengineering, water treatment, electronics, and optics. However, the nanomaterial-based methods have rarely been used in food science (specifically antioxidant research), probably because of the substoichiometric character and variable kinetics of the concerned reduction reactions of noble metal salts by antioxidants leading to metallic NP formation. When AgNPs are dispersed in liquid media, they exhibit a strong UV−vis absorption band that is not present in the spectrum of the bulk metal. This extinction band is attributed to collective excitation of the electron gas in the particles, with a periodic change in electron density at the surface (SPR, surface plasmon resonance absorption). Thus, nanoparticle-based TAC assays rely on the principle of chemical reduction of noble metal salts/acids by antioxidant polyphenols to produce noble metal NPs that can be easily identified/quantified via their localized SPR (i.e., charge density oscillations confined to metallic nanoclusters, abbreviated as LSPR) absorption. Most applications of NPs used as probes for food antioxidants are associated with the use of Au, Ag, magnetite (Fe_3_O_4_) or titania (TiO_2_) nanoparticles and quantum dots (QDs), in which chemical reduction-based nanotechnological colorimetric assays of antioxidant capacity make use of the formation or enlargement of noble metal nanoparticles, and antioxidants with the highest TAC values exhibited the highest ability to produce Au/Ag-NPs from Au(III)/Ag(I) salts, as recently reviewed by Apak et al. [13].

The LSPR absorption of noble metal nanoparticles has very high molar extinction coefficients (ε) and are normally expected to allow higher sensitivity in optical detection methods than conventional reagents. Nevertheless, it should be noted that the ε value for AuNP and Au-nanorod formation (at the order of 108 L mol^−1^ cm^−1^) is calculated with respect to the relative concentration of NPs among all metallic noble metal atoms, i.e., when HAuCl_4_ is reduced with a reducing agent such as sodium citrate or borohydride, the resulting AuNP clusters may contain up to several hundred thousands of gold atoms, constituting only a small fraction of total reduced Au-atoms. Consequently, the molar absorptivities (ε) and hence sensitivities of AuNP-based antioxidant assays toward reducing polyphenols are not extremely high but at the order of the ε values of other well-known spectrophotometric TAC methods. However, most of the existing NP-based TAC sensors have utilized the formation rather than the enlargement of noble metal NPs upon reaction with antioxidant compounds, and therefore may not yield concentration-dependent linear responses due to kinetic factors (i.e., NP size is dependent upon the reducing power of reducing agent; polyphenols and flavonoids display a wide range of redox potentials, and may thus show slow, medium or fast kinetics for reducing noble metal salts, giving rise to a wide range of sizes for noble metal NPs with maximal wavelength shifts for LSPR absorption). 

The working principle of antioxidant activity assays using noble metal NPs may be broadly classified under four categories: (i) formation and growth (enlargement), (ii) aggregation and agglomeration, (iii) disintegration, (iv) specific interaction of NPs [14], under the influence of antioxidant analytes. Certainly, there are some overlaps among these mechanistic classes. These mechanisms enable antioxidant quantitation to different extents of selectivity and sensitivity.

(i) In noble metal NP formation, antioxidants chemically reduce noble metal salts such as HAuCl_4_, AgNO_3_, H_2_PtCl_6_, RhCl_3_, PdCl_2_, etc. to produce the corresponding noble metal NPs in a dispersion. Antioxidants are identified and quantified with the aid of LSPR band wavelengths and intensities of the corresponding noble metal NPs, respectively. Because stronger antioxidants produce smaller NPs and LSPR band wavelength (λ_max_) is a function of particle size, a wide range of NP sizes will form from an antioxidant mixture, causing shifts in λ_max_ and subsequent difficulties in antioxidant quantitation. Starting with NP seeds with the use of borohydride or citrate, and then adding antioxidant analytes to the mixture will give rise to a more controlled enlargement of NPs, with little shifts in LSPR wavelengths (in this case, antioxidant concentrations can be found from the increase in LSPR absorbance). In NP formation-enlargement procedures upon reaction with antioxidants, all antioxidants having an appreciable reducing power will react with noble metal salts, and the assay will be totalistic (i.e., measuring TAC) rather than selective. However, from another viewpoint, this is also the strength of these assays, as the TAC parameter reflects the integrated collaborative action of all antioxidants in a mixture, which may be more beneficial in health issues than individual antioxidant concentrations. Under normal circumstances, although being simple and cost-effective, most of these TAC assays cannot effectively compete with classical robust electron transfer−based TAC assays [14] because NP formation and aggregation are adversely influenced by a great many physico-chemical factors.
(ii) In aggregation procedures, certain types of antioxidants (e.g., thiols) can form a self-assembled layer on noble metal NP surfaces, and with the aid of additional donor-acceptor or electrostatic interactions, these NPs can form aggregates, shifting the λ_max_ to longer wavelengths. These methods are generally more specific than conventional NP-based TAC assays. For example, an AuNPs aggregation-based spectroscopic method was devised for cysteine by Li et al. [15]. Through the covalent combination with the –SH group and the electrostatic binding with the –NH_3_^+^ group of cysteine, AuNPs can self-assemble to form a network structure, which results in greatly enhanced resonance light scattering and a bathochromic shift in the visible LSPR band. Li et al. [16] have demonstrated a highly sensitive and selective colorimetric detection method for cysteine using gold nanoparticles probes. This assay relies upon the distance-dependent optical properties of gold nanoparticles, the self-assembly of cysteine on gold nanoparticles, and the interaction of a 2:1 cysteine/Cu^2+^ complex. In the presence of Cu^2+^, cysteine could rapidly induce the aggregation of gold nanoparticles, thereby resulting in red-to-blue (or purple) color change. An LOD as low as 10 nmol·L^−1^ was achieved for cysteine, with good selectivity over other α-amino acids, glutathione, and thioglycolic acid.
(iii) Some turn-off LSPR sensors for hydrogen peroxide depend on the disintegration of AgNPs upon H_2_O_2_ degradation such that the test solution may turn colorless at the end [17]. These turn-off H_2_O_2_ sensors were synthesized from AgNPs stabilized by starch [18], PVA [19] and other polymers [20], but all of them seriously suffer from problems associated with linear response, because AgNPs act as a catalyst to degrade the analyte (H_2_O_2_), which, in turn, degrades the catalyst itself [17]. Due to the non-linear response of the sensor, antioxidants expected to inhibit AgNPs disintegration via H_2_O_2_ scavenging do not reasonably restore the LSPR absorbance signal in a concentration-dependent manner, which makes TAC estimation difficult.
(iv) Specific interaction sensors involve chemical reactions of antioxidant analytes with a modified noble NP surface, and are more specific by nature than noble metal NP formation sensors based on chemical reduction of the corresponding metal salt. Interaction sensors form a partially intersecting set with aggregation sensors mentioned in (ii). For example, additional linkages between thiol−assembled AuNPs to form higher aggregates with a concomitant shift of LSPR band wavelength is also a specific interaction capable of separately detecting thiol antioxidants. Displacement of surface-adsorbed dyes from noble metal NPs by preferential adsorption of thiols, followed by optical absorption or fluorescence measurement of the released dyes is another example of these types of sensors specific for thiol detection. A nano-colorimetric probe for ascorbic acid (AA) works with alkyne- and azide-functionalized AuNPs in that, when (AA + Cu^2+^) is added to the mixture dispersion, alkyne-azide click reaction (1,3-dipolar cycloaddition) takes place to result in AuNPs aggregation. This reaction is accelerated by AA acting as a reductant for Cu^2+^, and an AA concentration−dependent color change (from red to purple) is observed to achieve a LOD of 3 nmol·L^−1^ for AA [21].

By making use of the growth-enlargement mechanism, our group developed a AgNP based sensor (based on silver coating of pre-formed seeds of NPs upon reduction by polyphenols) for measuring the TAC level of complex food and biological samples with reasonable precision and accuracy [22]. We named this method as the Silver NanoParticle Antioxidant Capacity (SNPAC) assay. In this assay, Ag^+^ was first reduced to small-sized spherical AgNPs (SNPs) with the weak reductant trisodium citrate. Upon addition of polyphenolic antioxidants for TAC measurement, the preformed AgNP seeds were coated with newly formed nanoparticle layers in a controlled manner (thereby compensating for variable kinetic effects of the tested antioxidants having variable redox potentials), and the λ_max_ values at 423 nm did not shift with respect to different classes of polyphenols, because it was the outermost shell that was decisive on LSPR absorption (had it been initial nucleation, most polyphenols would have yielded significant shifts in LSPR-λ_max_). The absorbance increase with respect to the blank (of pre-formed seeds) varied linearly with polyphenol concentration, and thus, unlike its counterparts, the developed SNPAC assay showed reasonable precision and accuracy for measuring antioxidant levels of complex samples. The LOD for trolox was found to be 0.23 µmol·L^−1^ in the proposed method. The precision, which is expressed as the relative standard deviation (RSD, %) in the tested concentration range, was ~5.2%. The SNPAC absorbances of TR were linear within the concentration range of 1.28 × 10^−7^–1.12 × 10^−4^ mol·L^−1^ (as final concentrations in solution). Silver was preferred over gold in the growth of NPs, because a Ag colloid is expected to have a stronger and sharper plasmon resonance than Au, as its Mie resonance occurs at energies distinct from any bulk inter-band transition [23].

An ‘unconventional’ optical fiber reflectance sensor for catechin by immobilized 2,2′-(1,4 phenylenedivinylene) bis-8-hydroxyquinoline (PBHQ) on TiO_2_ nanoparticles (NPs) was developed by Apak et al. [24]. The working principle of the sensor was the formation of ‘indophenol blue’ dye on PBHQ-immobilized TiO_2_ nanoparticles as a result of *p-*aminophenol (PAP) autoxidation with dissolved O_2_ in alkaline medium. Among quercetin, rutin, naringenin, naringin, gallic acid, caffeic acid, ferulic acid, *p-*coumaric acid, catechin, epicatechin, epicatechin gallate, epigallocatechin, epigallocatechin gallate, and trolox, only catechin group antioxidants (tea catechins) delayed the color formation on nano-sized TiO_2_, measured via 710 nm-reflectance. Quantitative analysis was made by recording reflectance versus time, and the difference between the areas under curve (ΔAUC) in the presence and absence of catechin was linearly correlated to catechin concentration. The selectivity of the sensor for catechins was shown in tea infusions, and was ascribed to the nonplanar structure of catechin interfering with the formation of perfectly conjugated indophenol blue on TiO_2_ surface. The calibration curve (as ΔAUC vs. concentration) of CAT was constructed with a linear correlation coefficient close to unity (r = 0.98), with which the LOD for catechin was found to be 0.37 μmol·L^−1^ in the proposed method. 

Thiol-type (R-SH) antioxidants can be sensed by Au/Ag-NPs firstly by LSPR absorption wavelength shifts and secondly by displacement of fluorophores/chromophores upon functionalization of these nanoparticles with thiols, as the S-atom of thiols may form a strong covalent bond with Au. Our research group introduced a new optical sensor using Ellman’s reagent (DTNB)-adsorbed gold nanoparticles (AuNPs), abbreviated as DTNB-Au-NP, in a colloidal solution devised to selectively determine thiols from biological samples and pharmaceuticals. 5,5’-Dithio-bis(2-nitrobenzoic acid) (DTNB) as free thiol reagent was adsorbed through non-covalent interaction onto AuNPs, and the absorbance changes associated with the formation of the yellow-colored 5-thio-2-nitrobenzoate (TNB^2−^) anion as a result of reaction with biothiols was measured at 410 nm [25]. TNB as a thiol compound has a relatively low pKa and may easily be the leaving group in a thiol-exchange reaction on AuNPs. The AuNPs were initially synthesized by classical citrate reduction method [26] and then derivatized with DTNB. The DTNB-Au-NP thiol-sensor gave a linear absorbance response over a wide concentration range of standard biothiols comprising cysteine, glutathione (GSH), homocysteine, cysteamine, dihydrolipoic acid and 1,4-dithioerythritol. The DTNB-Au-NP sensor could be successfully applied to complex samples. Under optimized conditions, cysteine was quantified by the DTNB-Au-NP sensor, with a detection limit (LOD) of 0.57 μmol·L^−1^ and acceptable linear range from 0.4 to 29 μmol·L^−1^ (r = 0.998). 

Another noble metal NP-based sensor for the food preservative, nitrite, was developed for its colorimetric determination using 4-aminothiophenol (4-ATP) modified gold nanoparticles (AuNPs) and naphthylethylene diamine (NED) as coupling agent for azo-dye formation, abbreviated as “4-ATP − AuNP + NED” colorimetric method [27]. Our selective nitrite sensor utilized the interaction of LSPR absorption of Au-NPs through 4-ATP functionalization with diazo-coupling by naphthylethylene diamine on the NPs surface. The initial citrate-stabilized AuNP colloidal solution was red, turned colorless upon derivatization with 4-ATP, and the 4-ATP − AuNP + NED functionalized NPs gave a blue to blue-violet color with nitrite, showing bathochromic shifts in LSPR absorption. The LOD for nitrite was ≤2 μmol·L^−1^. Unlike its literature counterparts showing an absorbance decrease over a narrow range of nitrite concentrations, this sensor showed an absorbance increase over nearly one order-of-magnitude levels of nitrite, probably showing that a distinct dye product was formed on AuNPs conforming to Beer’s law (i.e*.*, had it been simple agglomeration of NPs, a wide linear concentration range could not have been observed).

A portable paper-based TAC nanosensor involved the reaction between antioxidants and gold nanoparticles on filter paper, in which gold (III) ions (AuCl_4_^−^) were impregnated [28]. The quantitation of antioxidant capacity was based on the direct reduction of Au(III) to Au(0) by antioxidant compounds on the paper. The intensity of the red color on paper was proportional to the reducing power and concentration of the tested antioxidant. The semi-quantitative sensor was scanned against a white background for color intensity analysis and the mean color intensity of sensing area image was analyzed by using IMAGE J software. The repeatability and reproducibility of the assay, expressed as the intra- and inter-day RSD, were 3–6% and 7–12%, respectively, for gallic acid levels between 0.05 and 1.0 mmol·L^−1^. The accuracy of the method, expressed as recovery of gallic acid (within the range of 0.01−0.1 mmol·L^−1^) from various tea infusions, varied between 85% and 108%. 

#### 1.1.3. Electrochemical Sensors for Detecting Biomarkers of Oxidative Stress and Antioxidants

Electrochemical devices have simple and inexpensive equipment, and related methods depend on electron transfer reactions [29]. The design principles of electrochemical biosensors as relevant tools of antioxidant activity assessment have been excellently reviewed in literature [29,30,31,32]. The strategy is based on electrochemical measurement of oxidative damage produced on lipids, proteins or DNA, and restoration of their original electrochemical signals in the presence of protective antioxidants. Most electrochemical sensing approaches focus on cytochrome *c*, superoxide dismutase and DNA probes [30]. Since ROS may cause oxidation on DNA bases and on deoxyribose moieties, these disadvantages can be used for modelling ROS damage in human body. For this purpose, different DNA-based sensors were developed and used for evaluation of total antioxidant capacity of certain samples. The most widely used technique is immobilization of DNA (especially double strand DNA, dsDNA, from calf thymus) on screen printed carbon electrodes using simple adsorption techniques. In this method, mostly guanine oxidation peak was detected between +0.8 and +1.0 V against a Ag/AgCl electrode with square wave voltammetry (SWV). To obtain ROS, a Fenton solution was used into which the electrode was dipped, and then antioxidants were added into this solution. In the proposed system, the intensity of peak current and guanine concentration were proportional, where the presence of Fenton-generated ^•^OH caused a decrease in the signal and radical scavengers (antioxidants) caused an increase in the peak current intensity again. So TAC could be determined using this method [33,34]. Electrochemical labelling which can interact with dsDNA is also another method to measure DNA damage. This interaction means an intercalation or an interaction depending on the ionic strength. In the proposed method, redox labeled DNA layers could be measured by differential pulse voltammetry (DPV). A decrease in the measured electrochemical signal was proportional to the amount of ROS in the reaction system and antioxidants protected the signal [35,36]. Bučková et al. applied this method to detect TAC in yeast polysaccharides using as a redox marker, namely 1,10-phenanthroline cobalt(III) complex, [Co(phen)_3_]^3+^ [37]. In another study, Qian et al. covalently attached dsDNA on poly(amidoamine) (PAMAM)-encapsulated Au-Pd/chitosan composite [38].

Since DNA adsorbed on modified sensor electrodes displayed slow electron-transfer kinetics, electron shuttles and redox mediators (such as NADH, methylene blue, Ru(III)- and Co(III)-bipyridyl complexes, etc.) were generally applied to speed up electrochemical reactions. The oxidation peak currents of suitable DNA bases such as adenine or guanine [34] may be measured (e.g., with the aid of a biosensor consisting of dsDNA immobilized on a screen-printed electrode) to evaluate the DNA hazard produced by hydroxyl or superoxide radicals and its subsequent restoration in the presence of protective antioxidants acting as radical scavengers. Kamel et al. immobilized purine bases on a glassy carbon electrode to manufacture a biosensor and evaluate the TAC of flavored waters. The mechanism of the biosensor involved monitoring of the changes of the intrinsic anodic response (by the electrochemical technique of SWV) of the surface-confined guanine and adenine nucleic bases, resulting from their interaction with ROS—derived from Fenton-type reactions—in the absence and presence of tested antioxidants (ascorbic acid being used as standard) [39]. The ROS scavenging properties of antioxidants deactivated radicals such as ^•^OH and protected nucleic bases from oxidative damage. The strong interaction of adenine and guanine layers with the electrode surface made the confined biosensor a useful device for TAC measurement of beverages with high reproducibility and constant sensitivity. The most commonly used oxidation biomarkers of damaged DNA are 8-hydroxydeoxyguanosine (8-OH-dG) and purine bases: guanine and adenine. Zhang et al. made use of DNA adsorption on electrochemically modified GCE. During oxidation, ^•^OH was generated by a Fenton reaction (i.e., from Fe(II) + H_2_O_2_), whereas ascorbic acid (AA) and mannitol were added as radical scavengers. The reaction mixture was dropped to the modified GCE, and the oxidation peaks of adenine, guanine and 8-OH-dG were recorded [40]. In a similar study, a purine biosensor was used to estimate the TAC of beverages with SWV. In the study, adenine or guanine was electrochemically immobilized on GCE (i.e., an adsorptive accumulation step was used) and the resulting peak current due to the oxidation of nucleic bases was measured [41]. Mello et al. manufactured a DNA-electrochemical biosensor for investigating antioxidative properties of different plant materials by immobilizing a dsDNA layer on a screen printed electrode and using a Fenton reaction to oxidize DNA. To oxidize the immobilized calf thymus DNA, the electrode was dipped into iron(II)-EDTA reaction mixture and oxidation was initiated by H_2_O_2_ addition. Guanine oxidation peak was followed by the SWV technique. Fenton reaction was repeated in the presence and absence of antioxidant samples, and the observed voltammetric changes were evaluated [34].

**Figure 3:**
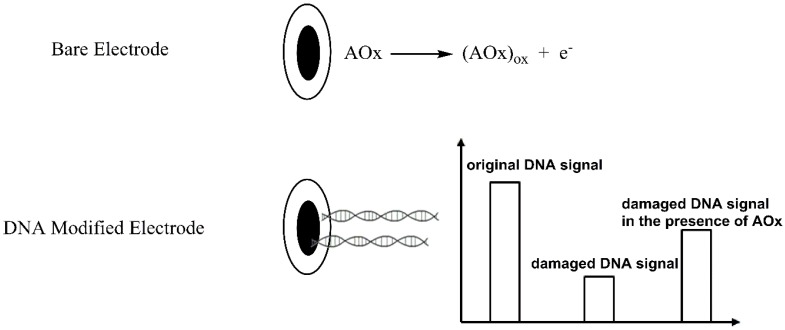
Electrochemical sensing of oxidative DNA damage and its restoration by antioxidants (immobilization method on the working electrode was adsorption on GCE).

Barroso et al. proposed a method depending adenine-rich oligonucleotide (dA21) as probe, adsorbed on carbon paste electrodes for the assessment of the antioxidant capacity. In the study, the oxidation product of adenine, produced by a Fenton reaction, acted as a catalyst in the oxidation of NADH. The increase in the electrocatalytic current was measured by differential pulse voltammetry (DPV). The proposed method was used to determine the TAC of certain beverages and ascorbic acid [31]. The main principle of electrochemical antioxidant sensing by DNA-modified electrodes is shown in Figure 3, and the analytical performances of a number of DNA-modified electrodes exposed to various radicals are compared in Table 2. 

Another strategy for electrochemical detection of oxidative damage on biomarkers is to immobilize proteins (bovine serum albumin: BSA, hemoglobin, etc.) on modified electrodes. For this purpose, BSA was adsorbed onto a GCE surface, and Co(bpy)_3_^3+^ was used as a redox indicator to monitor BSA damage induced by hydroxyl radicals produced from a Fe^2+^/H_2_O_2_ reaction [42]. Intact BSA gave a good oxidative peak due to the oxidation by Co(bpy)_3_^3+^, and after oxidation of BSA by the Fenton system, the peak due to the Co(III)-complex showed a sharp decrease, which was restored by antioxidants (ascorbic acid, catechin, etc.). In other studies using the same probe, BSA/poly-*o*-phenylenediamine (PoPD)/carbon-coated nickel (C-Ni) nanobiocomposite film modified glassy carbon electrode (BSA/PoPD/C-Ni/GCE) was developed. BSA was subjected to Fenton oxidative damage, and a decrease in the PoPD oxidation signal intensity was taken as an indicator of BSA damage, where antioxidants restored the signal [43,44].

### 1.2. Colorimetric Sensors and Nanoprobes with Miscellaneous Mechanisms for Antioxidant Detection

There are other colorimetric sensors and nanoprobes for antioxidant assessment that cannot be classified under a unique mechanism. Portable ceria nano-particle based sensor (NanoCerac) assay was developed by Sharpe et al. [48] for rapid and sensitive detection of food antioxidants. The sensor was fabricated of immobilized ceria nanoparticles on filter paper by surface adsorption between the –OH rich ceria surface and the cellulosic fibers [49]. For example, CeO_2_ reacted with ascorbic acid to yield Ce(III)-oxide and dehydroascorbic acid. As antioxidants partly reduced Ce(IV) to Ce(III), possibly a mixed-valent oxide was formed and the blue color of the sensor was related to the binding affinity and electron donating ability of antioxidants to ceria on filter paper. Sensors were scanned and processed by Adobe Photoshop program. The colorimetric response was concentration− dependent, with detection limits ranging from 20 to 400 mmol·L^−1^ depending on the antioxidant involved.

In general, transition metal oxide nanoparticles impregnated on filter paper proved to be effective probes for quantifying polyphenolic antioxidants. Multiple metal oxide nanoparticles with varying polyphenol binding behavior were used as active sensing materials to devise sensor arrays. The principle of polyphenol detection/quantitation was essentially polyphenol binding coupled to charge-transfer interactions. While CeO_2_ and Fe_2_O_3_ formed a brown color on the paper in the presence of antioxidants, titanyl oxalate formed a bright orange complex, TiO_2_, ZrO_2_ and SiO_2_ a yellow-green and ZnO a bright yellow color [48]. As the highest sensitivity was achieved with transition metal oxides, the authors reported a LOQ of 0.04 mmol·L^−1^ for gallic acid using nanoceria on paper. Unique colors were formed as a result of charge-transfer (in the visible range) between specific HOMO-LUMO levels of chemically interacting species, i.e., polyphenol donor and transition metal oxide acceptor. As these types of sensors may not reach very low detection limits for antioxidants, an alternative strategy to increase the sensitivity of these transition metal oxide sensors is the use of redox mediators as auxiliary ligands or probes. For example, oxidase-mimic nanoceria may cause the oxidation of a peroxidase substrate redox dye to a colored product which partly bleaches with antioxidants in a concentration-dependent manner.

Palaroan et al. [50] reported an optical fiber chemiluminescence (CL) biosensor for testing antioxidants, utilizing luminol and hematein co-immobilized on a cellulosic membrane disc. Antioxidant capacity of sample was quantified via quenching of CL produced from the reaction of H_2_O_2_ with luminol in the presence of hematein as catalyst. Under the experimental conditions, H_2_O_2_ generated hydroxyl radicals which in turn induced the CL reaction of luminol. Antioxidants, when present, scavenged ^•^OH and inhibited the CL signal. The sensor response (of CL quenching) varied linearly with the logarithm of antioxidant concentration over three orders-of-magnitude. The LOD was reported as 100 nmol·L^−1^ for propyl gallate and ascorbate.

A molecular imprinted polymeric microspheres (MIPm)-based flow injection chemiluminescence (FI-CL) sensor was developed for sensitive quercetin determination [51]. Adsorption of quercetin took place via hydrogen bonds between its –OH, =O groups and the –NH_2_, =O groups of MIPm. As the alkaline luminol-H_2_O_2_ reagent is not selective for quercetin, the real sample solution containing quercetin was let to pass through two adsorbent tubes, one containing MIPm and the other non-molecularly imprinted polymeric microspheres (NIPm), and the difference in CL emission was taken as a specific indication of quercetin. The linear concentration range was 1.4−160 μmol·L^−1^, RSD ≈ 3%, and LOD 0.9 μmol·L^−1^ quercetin.

**Figure 4:**
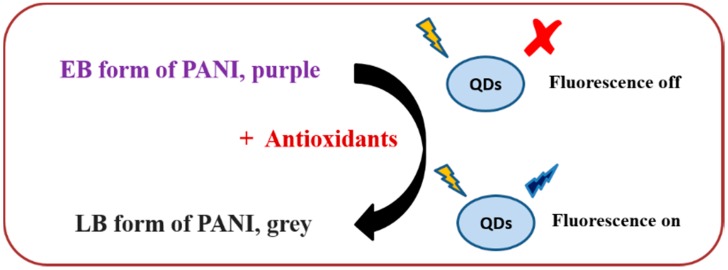
Scheme of the fluorescent turn-on sensing strategy for antioxidants.

A simple method for antioxidant assessment based on the redox transformation of polyaniline (PANI) was reported by Li et al. [52]. PANI is a widely used conducting polymer, and the emeraldine base (EB) of PANI fibers can be reduced to leuco-emeraldine base (LB) form in the presence of antioxidants, giving rise to color changes. The absorbance of PANI at 580 nm decreased by adding increasing concentrations of antioxidant and the color of solution gradually changed from purple to blue and to light gray. In addition, EB form of PANI could efficiently quench the fluorescence of CdTe quantum dots, and the fluorescence was recovered with antioxidant addition (Figure 4). Quantum dots (comprising CdS, CdSe, CdTe, ZnSe, InAs, InP, etc.) are a special class of semiconductor nanocystals (of 2–6 nm diameter) playing an important role as fluorophores. They have size-tunable fluorescence, broad excitation spectra, and are especially useful for detecting thiol-type antioxidants. The fluorescence intensity of CdTe QDs (λ_max_ = 579 nm) increased linearly with added antioxidant concentration in the presence of EB form of PANI. The detection limits for glutathione and ascorbic acid were estimated to be 50 nmol·L^−1^ and 100 nmol·L^−1^, respectively.

A switch-on fluorescence sensor for glutathione (GSH) detection in food samples was recently reported by Xu et al. [53]. Graphitic carbon nitride quantum dots (g-CN QD)-Hg^2+^ chemosensor was used in the system, the initial fluorescence signal of which was quenched by Hg^2+^. However, upon the successive addition of GSH, this FL intensity was remarkably recovered due to GSH binding to Hg^2+^ which was subsequently released from the surface of quantum dots. Thus, in the presence of GSH, GSH and Hg^2+^ showed a competitive affinity to the functional groups on the surface of g-CN QDs, thereby switching the fluorescence sensor to the “on” state. The g-CN QD-Hg^2+^ chemosensor was claimed to have important advantages such as high selectivity, sensitivity, cost-effectivity and rapidity. Under optimal conditions, the linear concentration range was 0.16–16 μmol·L^−1^, with a LOD of 37 nmol·L^−1^ for GSH. The repeatability was 5.3% for standards and foods. 

Ultrasensitive detection of polyphenols using CdTe QDs as optical labels was reported by Akshath et al. [54]. After catalytic oxidation with laccase enzyme and O_2_, polyphenols were converted to mono/polyquinones which could quench QDs fluorescence. The quenching of CdTe QDs fluorescence was linear with respect to polyphenol concentration (λ_ex_ = 450 nm and λ_em_ = 582 nm). It was possible to determine polyphenols in a wide linear range, with a limit of detection (LOD) of 1 ng mL^−1^ and determination coefficient R^2^ = 0.9702. Using the same type of quantum dots, an automated CL assay was recently developed for the TAC determination of food samples, based on luminol oxidation by in-line photogenerated radical species from l-glutathione-capped CdTe QDs [55]. Antioxidants capable of scavenging the generated free radicals prevented the reaction of ROS with luminol, quenching the CL analytical signal. The authors reported an analytical linear response range between 0.1 and 5 µmol·L^−1^ of trolox and a LOD of 0.05 µmol·L^−1^. 

A triple-dimensional sensing chip, composed of CdSe/ZnS QDs and graphene, was developed to discriminate eight antioxidants, based on simultaneous utilization of FL, ECL and MS properties of the nanocomposites [56]. Of the plausible mechanisms, the authors focused on the number of phenolic hydroxyl groups on the analytes so as to be adsorbed on the sensing nanochip, causing fluorescence quenching of the QDs. Unknown samples randomly taken from the training set of antioxidants at concentrations of 0.7 μmol·L^−1^ were successfully classified by principal component analysis (PCA) with recoveries of 92.5%. A useful property of QDs for antioxidant activity measurement is their photobleachability induced by ROS (e.g., generated by UV radiation) which can be reduced in the presence of antioxidants. 

Wang and coworkers proposed a graphene oxide amplified electrogenerated chemiluminescence (ECL) of quantum dots platform [57] for the selective sensing of glutathione (GSH) among other thiol-containing compounds (e.g., cysteine and oxidized glutathione). The ECL intensity of graphene oxide amplified QDs platform showed a rapid decrease (e.g., at 120 μmol·L^−1^ GSH) while CdTe QDs (without graphene) showed no changes with increasing GSH concentration. This platform showed a LOD of 8.3 μmol·L^−1^ and selective quantification within the linear range of 24 to 214 μmol·L^−1^ GSH. 

Zhao and coworkers recently developed titania nanotubes (TiNTs)−functionalized indium tin oxide (ITO) coated glass for flow injection analysis (FIA) to intensify the electrochemiluminescence (ECL) of luminol for sensitive detection of ROS and antioxidants [58]. Under optimal conditions, a linear concentration range of 0.1–30 mg L^−1^ and a detection limit of 1.65 × 10^−10^ g for resveratrol was obtained by using ECL of luminol on functionalized ITO in FIA mode. TiNTs were assumed to act as an effective oxygen carrier, and the catalyst for oxygen transformation owing to its large specific surface area. As a result of a series of Haber-Weiss reactions, singlet oxygen was produced from the reduction products of O_2_, i.e., H_2_O_2_, superoxide and hydroxyl radicals. TiNTs accelerated the formation of singlet oxygen from oxygen reduction and oxidation of luminol, resulting in enhanced ECL emission of luminol. 

## 2. Colorimetric Sensors and Nanoprobes for Detecting Reactive Species (ROS/RNS) and for Determining Scavenging Activity of Antioxidants

### 2.1. Hydrogen Peroxide

#### 2.1.1. Spectroscopic Sensors and Nanoprobes

Hydrogen peroxide (H_2_O_2_) is an important ROS formed in tissues through oxidative processes. H_2_O_2_ is produced by different oxidases and decomposed by catalases. Thus, the activity of these enzymes can be determined by monitoring the production or consumption of H_2_O_2_ [59].

**Scheme 1:**
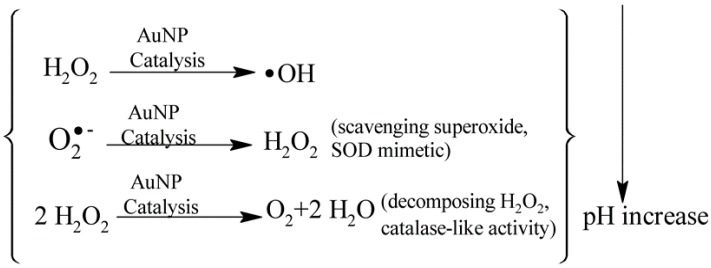
Schematic diagram of catalytic properties of AuNPs in decomposing H_2_O_2_ and scavenging superoxide anion radical.

In the presence of H_2_O_2_, AuNPs can elicit the generation of hydroxyl radicals in acidic environment and the production of oxygen under alkaline conditions. These interesting phenomena may be attributed to: (i) catalytic properties of AuNPs (such as catalase-like activity for the decomposition of H_2_O_2_) and (ii) the pH dependent physical and chemical properties of H_2_O_2_ (Scheme 1) [60]. 

There are methods in literature, based on the growth process of gold nanoshells (GNSs) [61,62], used to investigate the H_2_O_2_-scavenging activity of several phenolic acids and herbal extracts. Hydrogen peroxide induces GNSs formation by reducing [AuCl_4_]^−^ to Au, deposited on the surface of SiO_2_/AuNP composite with subsequent enlargement of AuNPs to form a continuous shell layer, and antioxidants, when present, inhibit this growth by their H_2_O_2_ scavenging action. During the H_2_O_2_-induced formation process of GNSs, their LSPR band shows large bathochromic shifts (i.e., to longer wavelengths) across the visible region, rendering analytical wavelength selection difficult. Phenolic acids, as well as tea and herb samples with H_2_O_2_-scavenging ability, prevents complete GNSs formation and their bathochromic shift. AuNPs on SiO_2_ cores were enlarged by increasing concentrations of H_2_O_2_, concomitant with spectral changes corresponding to SPR absorption bands of AuNPs, and antioxidants restricted H_2_O_2_-mediated formation of GNSs, enabling the determination of their scavenging activity. The authors defined the concentration of each antioxidant to cause a 50% wavelength inhibition ratio as IC_50_, where the IC_50_ values of salicylic acid (not classified as an antioxidant) and ferulic acid were 470 and 8 µmol·L^−1^, respectively.

Ma et al. tested some phenolic acids to reach the conclusion that caffeic acid was the strongest H_2_O_2_-scavenger whereas *trans*-cinnamic acid was the weakest [62]. The H_2_O_2_ scavenging activity of phenolic acids was correlated to the substitutional pattern in the aromatic ring and the degree of hydroxylation. Chen and coworkers developed a method for assessing H_2_O_2_ scavenging activity with the use of SiO_2_/AuNPs as SERS nanoprobes, where H_2_O_2_ reduced Au(III) to Au(0) mediating the growth of these nanoshells and subsequent gold coverage of silica cores, and antioxidants caused a drop in SERS-activity by preventing the H_2_O_2_-mediated growth of SiO_2_/AuNPs [63]. The surface coverage of AuNPs on silica cores increased with increasing H_2_O_2_ concentration, whereas antioxidants like tannic acid (with LOD ≤ 5 µmol·L^−1^) restricted gold nanoshell formation and the resulting SERS activity to a greater extent than L-malic acid having less –OH groups. 

AgNPs have the ability to reduce ROS with Ag(0)-Ag(I) oxidation, resulting in the decomposition of AgNPs and a decrease in LSPR absorption. As the standard reduction potentials for the H_2_O_2_/H_2_O and O_2_/H_2_O_2_ redox couples are 1.76 V and 0.70 V, respectively, and for Ag^+^/Ag^0^ 0.80 V, it is thermodynamically feasible for hydrogen peroxide to act both as an oxidizing agent for AgNPs and a reducing agent toward Ag^+^ ions. This provides an opportunity for Ag^0^NPs−based determination of hydrogen peroxide.

AgNPs presumably form a strongly oxidizing intermediate complex with H_2_O_2_. The generation of O_2_^•−^ is initiated by the H_2_O_2_-mediated oxidation of AgNPs [64]. The reactivity of AgNP toward H_2_O_2_ decreases with decreasing pH, while at high pH, reformation of AgNPs by a back reaction (from Ag^+^ and O_2_^•−^) was essentially complete [65]:

AgNP + H_2_O_2_ → intermediate

Intermediate + H_2_O_2_ → Ag^+^ + O_2_^•–^ + H_2_O

There is another way which is believed to lead to the generation of O_2_^•−^, the oxygen mediated oxidation of AgNPs [66]:

AgNP + O_2_ → Ag^+^ + O_2_^•–^

Hydrogen peroxide detection methods based on the catalytic degradation of AgNPs are more common in literature due to the favorability of the concerned redox potential [18,19,20], but measurements based on NP degradation may suffer from reproducibility problems. Nevertheless, this type of sensor—based on the decomposition of H_2_O_2_ resulting in the degradation of AgNPs, with subsequent changes in the refractive index of the nanocomposite layer and a shift in LSPR wavelength—was claimed to show high sensitivity and a wide linear range (between 10^−8^ and 10^−1^ mol·L^−1^), fast response, and good applicability to remote and online sensing of H_2_O_2_ [67]. On the other hand, a rare study concerning SPR signal amplification of AgNPs relied on the reductive ability of H_2_O_2_ toward Ag(I) for H_2_O_2_ quantitation in the micromolar range. In this assay, Ag^+^ was reduced to Ag^0^ by O_2_**•**^−^ generated via UV radiation−aided decomposition of H_2_O_2_ in alkaline media. As the conversion of glucose to gluconic acid is catalyzed by glucose oxidase accompanying the formation of H_2_O_2_, both H_2_O_2_ and glucose in blood could be determined [68]. This method could determine H_2_O_2_ with concentrations between 5.0 × 10^−7^ and 6.0 × 10^−5^ mol·L^−1^, with a LOD of 0.5 µmol·L^−1^.

#### 2.1.2. Electrochemical Nanosensors

As direct electrochemical detection of H_2_O_2_ requires a high potential around 0.6 V at which other oxidizable species such as ascorbic acid, uric acid, and acetaminophen interfere [69], sensor electrodes are manufactured to detect the analyte at lower potentials, e.g., in the presence of AgNPs catalyzing the reduction of H_2_O_2_ [70]. Zhao et al. [69] developed an electrochemical H_2_O_2_ sensor based on multi-wall carbon nanotube/silver nanoparticle nanohybrids (MWCNT/Ag nanohybrids)‒modified gold electrode; the reduction current in this electrode was larger than that of MWCNT‒modified electrode in the presence of H_2_O_2_. Similar measurements were also carried out with MWCNT/Au electrode, but the obtained signals were found to be too low (probably due to the fact that O_2_/H_2_O_2_ and Ag^+^/Ag^0^ reduction potentials were quite close). Most electrochemical sensors perform H_2_O_2_ detection at around 0.6 V, but unfortunately, this potential is open to interference by other electro-active compounds. Thus, Zhao et al. [69] chose a voltage of −0.2 V to remove interferences, owing to the catalytic activity of their modified electrode. At the specified potential, the sensor gave a linear response between 0.05 and 17 mmol·L^−1^, with a LOD of 0.5 μmol·L^−1^ for H_2_O_2_. 

Cui et al. [70] developed a novel strategy to fabricate a H_2_O_2_ sensor electrode by electrodepositing AgNPs on a glassy carbon electrode modified with three-dimensional DNA networks. The electrochemical sensor had a favorable catalytic ability for the reduction of H_2_O_2_. The DNA network was claimed to prevent agglomeration and allow homogeneous deposition of AgNPs onto the electrode surface, resulting in an increase in the catalytic effect of the electrode in H_2_O_2_ detection. The linear concentration range of the AgNPs/DNA/GC electrode was 4 μmol·L^−1^–16 mmol·L^−1^ with a detection limit of 1.7 μmol·L^−1^; minimal interference was seen from electroactive substances like ascorbic and uric acid [70].

Redox mediators have been used to decrease the overpotential and enhance the electron transfer kinetics of H_2_O_2_ [71]. The glassy carbon electrode was modified with cobalt oxide nanoparticules in pH 7 phosphate buffer (PBS). The resulting Co_3_O_4_-NPs/GC electrode showed excellent electro-catalytic activity for hydrogen peroxide. The contribution of Co_3_O_4_ nanoparticles to electrode performance comprised decreased overpotential and accelerated electron transfer kinetics. The electrode was used for determination of H_2_O_2_ via cyclic voltammetry within the potential range of 0.4 V–1.0 V in pH 7 PBS. A linear response was obtained from 1 to 9 µmol·L^−1^, and the detection limit was 0.05 µmol·L^−1^ at the anodic potential of 0.75 V. Rotated (at 2000 rpm) Co_3_O_4_-NPs/GC electrode was also used for amperometric determination of H_2_O_2_, enabling a linear calibration range of 4–80 nmol·L^−1^ and a LOD of 0.4 nmol·L^−1^ at 0.75 V.

Although enzyme-immobilized sensor electrodes have also been used for electrocatalytic H_2_O_2_ determination, these enzymes are quite expensive and may lose activity during measurement. Noble metal (Au, Ag, Pt, Pd) nanoparticles have been suggested as electrocatalysts for sensing H_2_O_2_ due to their larger specific surface area, excellent conductivity and electrocatalysis. The analytical performances of some electrochemical nanosensors were compared in Table 3.

### 2.2. Superoxide Anion Radical

#### 2.2.1. Spectroscopic Sensors and Nanoprobes

Superoxide anion radical (O_2_^•–^) is the precursor of hydrogen peroxide, singlet oxygen and hydroxyl radical in the body which causes cell damage by initiating the peroxidation of the membranal lipids [91]. Most common techniques used for detecting superoxide anion radicals are electron spin resonance (ESR) spectroscopy, chemiluminescence, fluorescence spectroscopy and electrochemical detection. Among these methods, fluorescent probes show high sensitivity and selectivity. Hydroethidine (HE) has been known as a superoxide radical probe by measuring the fluorescence of ethidium (E^+^) thought to be its characteristic reaction product. Carbon-dots (CD) conjugated with HE were used as selective ratiometric fluorescent sensors for biosensing superoxide radical in living cells [92]. The ratio of the fluorescence emission intensity monitored at 610 nm in the presence of increasing concentration of O_2_^•–^ to the constant emission intensity of CD-HE probe, observed at 525 nm, was evaluated to compensate for background fluorescence. This fluorescent probe exhibited lower LOD (~100 nmol·L^−1^) in comparison to other O_2_^•–^ probes [93,94] and remained independent of pH in neutral-to-weakly alkaline solution. However, the reaction rate decreased as the rate of O_2_^•–^ production increased, probably owing to the catalytic effect of HE on the dismutation of O_2_^•–^ [95]. 

Li et al. designed a selective fluorescent nanoprobe to eliminate fluorescence signals of other reactive oxygen species. 2-Chloro-1,3-dibenzothiazolinecyclohexene (DBZTC) was loaded to Ag@SiO_2_ core/shell nanoparticles; 3-aminopropyltriethoxysilane (APTES) was added to these core/shell NPs to enable amine binding to the silica surface. Enhancement of fluorescence emission of DBZTC was related to the silica shell thickness, and this highly specific recognition of O_2_^•–^ provided 1000-fold stronger selectivity over H_2_O_2_ in living cells [96].

A ready-to-use fluorometric biosensor for detecting O_2_^•–^ was developed [97], based on the release of H_2_O_2_ which reacted with nonfluorescent Amplex Red (*N*-acetyl-3,7-dihydroxyphenoxazine) probe in the presence of horseradish peroxidase (HRP) enzyme to produce the fluorescent compound resorufin (λ_ex_ at 563 nm; λ_em_ at 587 nm). Amplex red was used for the fluorometric estimation of trace H_2_O_2_ [98]. SOD and HRP enzymes and the probe were immobilized at the same time to SiO_2_ sol-gel glass slides. The submicromolar concentrations of O_2_^•–^ were detected and LOD value was measured close to 20 nmol·L^−1^ (linear range up to 6 μmol·L^−1^ of xanthine). Although this sensor may be satisfactory for superoxide anion detection in phospholipid model membranes, foods, and drinks, it should be borne in mind that its measurement principle is based on H_2_O_2_ detection after superoxide dismutation, and therefore it may not be useful in biological media because the steady-state concentration ratio of H_2_O_2_/superoxide is around 100:1 in the mitochondrial matrix and 1000:1 in the cytosol [96]. 

Cytochrome *c* (Cyt *c*) is a highly water soluble small protein in cellular structure. O_2_^•–^ could be measured with the aid of reduction of Cyt *c* according to the given reaction:

Cytochrome^3+^*c* + O_2_^•–^ → Cytochrome^2+^*c* + O_2_

A selective and sensitive surface-enhanced Raman scattering (SERS)‒based nanosensor was performed using oxidized cytochrome *c*‒functionalized AuNPs [99]. By observing the differences between the SERS spectra of oxidized and reduced forms of Cyt *c*, O_2_^•–^ could be indirectly quantified by estimating cytochrome^2+^ *c*. The developed nanosensor could be delivered to living HeLa and human liver cells, enabling O_2_^•–^ estimation in real time noninvasively in biological systems. However, the reduction reaction of cytochrome^3+^ *c* is nonspecific because of the natural interference by many other reducing compounds such as Vitamin C or other antioxidants [100]. 

In another study, a fluorescent probe was developed utilizing the conjugation of oxidized Cyt *c* with negatively capped CdSe/ZnS quantum dots [101]. QD-oxidized Cyt *c* system proved to be an effective probe to detect superoxide anion radical where the fluorescence of QDs was enhanced significantly. The partially lost fluorescence intensity of the QD-oxidized Cyt *c* conjugate (relative to that of negatively capped CdSe/ZnS QDs) was significantly restored in the presence of O_2_^•–^ acting as a reductant toward Cyt *c*, which can be used to sense superoxide radical in HeLa and human liver cells. This probe did not interfere with other biologically relevant substances and ROS, except l-glutathione (GSH) which is present in the concentration ratio of 1000-fold O_2_^•–^. 

A novel fluorescent nanoprobe [102] was developed via immobilization of fluorescein-labeled hyaluronic acid, end-capped with dopamine to the surface of AuNPs. Hyaluronic acid (HA) is a natural polymer which can be used to detect hydroxyl and superoxide anion radicals. Catechol group in dopamine provides more stable immobilization of HA to AuNPs in intracellular environment. When HA was end-capped with thiol molecules unlike dopamine, background fluorescence intensity was enhanced by increasing concentration of glutathione present in intracellular medium. The working principle of the sensor is cleavage and fragmentation of the HA chains immobilized on the surface of the AuNPs by intracellular ROS, resulting in strong fluorescence-recovery signals proportional to the concentrations of O_2_^•–^ and ^•^OH. This probe was stable and sensitive for ROS in a highly reducing environment, but may be less selective than other similar NP-probes for ROS detection [99,103]. In the presence of ROS, the probe showed strong fluorescence intensity and lower detection limits (0.3 µmol·L^−1^ for O_2_^•–^, 1 µmol·L^−1^ for ^•^OH) among other reactive species such as H_2_O_2_, ONOO^–^, OCl^–^. In this study, ROS scavenging capacities of various antioxidants including ascorbic acid, *p*-coumaric acid, quercetin and α-tocopherol were evaluated by monitoring the decrement of fluorescence belonging to the nanoprobe, where α-tocopherol exhibited the highest scavenging activity due to its hydrophobic character providing permeability through cell membranes. 

A novel ratiometric fluorescent sensor for superoxide anion detection was developed with suffocated polystyrene nanoparticles (PS-SO_3_H)@terbium (Tb)/guanine (G) nanoscale coordination polymers (PS-SO_3_H@Tb/G NCPs). The fluorescence of PS-SO_3_H was slightly weakened as a reference, and the fluorescence of Tb^3+^ was largely weakened in the presence of O_2_^•–^, resulting in a selective ratiometric fluorescent sensor toward O_2_^•–^. The ratio of fluorescence intensity linearly decreased with increasing O_2_^•–^ concentration in the range of 10 nmol·L^−1^–6 µmol·L^−1^ with a detection limit of 3.4 nmol·L^−1^ [104].

Advantages/disadvantages and analytical performances of some nanoprobes and sensors for spectroscopic detection of O_2_^•–^ are listed in Table 4.

#### 2.2.2. Electrochemical Nanosensors

Xanthine/xanthine oxidase (XOD)−generated superoxide radicals can be effectively detected by electrochemical sensors with the aid of the following reactions:

$$\mathrm{xanthine}+H_{2}O+O_{2}\overset{\mathrm{XOD}}{\to }\mathrm{uric}\;\mathrm{acid}+2H^{+}+{O_{2}}^{•-}\;2H^{+}+2{O_{2}}^{•-}\overset{\mathrm{SOD}}{\to }H_{2}O_{2}+O_{2}$$

These reactions describe the catalytic dismutation of superoxide radical to hydrogen peroxide and oxygen by superoxide dismutase (SOD) on an electrode surface. The first-generation O_2_^•−^ biosensors were based on the direct measurement of the electrochemical response from the enzymatic product (H_2_O_2_) at the electrode [105]. The second-generation O_2_^•−^ biosensors involved specific ‘redox mediators’ that were able to shuttle electrons between the electrode and the reactive sites of SOD [106]. The third-generation O_2_^•−^ biosensors exploited direct electron transfer between the redox sites of the enzyme (i.e., SOD-Cu(I,II) sites responsible for redox cycling) and the electrode without any mediator [107,108].

Thiol (e.g., cysteine) self-assembled on gold (or AuNPs-modified) electrodes may be used as electron transfer promoters in this regard. For example, a third-generation biosensor for O_2_^•−^ was fabricated by immobilizing SOD on such an electrode (i.e., SOD/cysteine/Au electrode) to record the enhanced anodic and cathodic current responses resulting from the oxidation and reduction of O_2_^•−^, respectively, where the enhanced response was mediated by the SOD confined on the electrode [107]. The working potentials of the polarized electrode were suitable to eliminate interferences arising from physiological levels of H_2_O_2_, ascorbic and uric acids, and neurotransmitter metabolites. 

**Figure 5:**
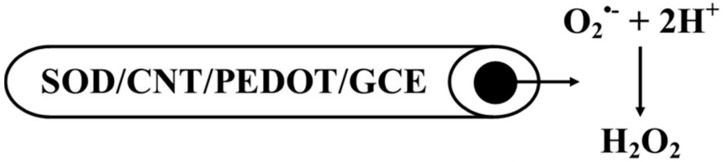
Schematic presentation of SOD biosensors based on PEDOT and CNT.

Braik et al. [109] developed a novel electrochemical biosensor for the direct determination of O_2_^•−^ using SOD enzyme. This biosensor consisted of multiwalled carbon nanotubes (MWCNT) together with the conducting polymer poly(3,4-ethylenedioxythiophene) (PEDOT) in different configurations (PEDOT/CNT/GCE and CNT/PEDOT/GCE) (Figure 5). 

The biosensor with carbon nanotubes (CNT) on top of PEDOT layer gave optimal analytical performance and was applied to the determination of the TAC value of beverages. The determination of superoxide at SOD/CNT/PEDOT/GCE was performed using fixed potential amperometry in neutral PBS buffer (LOD = 1 μmol·L^−1^). Superoxide radical anion detection is based on the enzymatic redox mechanism of SOD which can catalyze the dismutation of O_2_^•−^ to O_2_ and H_2_O_2_ via a redox cycle of the (Cu^I^/Cu^II^) redox couple in Cu–Zn SOD. 

The reactions involved in this catalytic redox cycle are as follows:

SOD (Cu^+^) + O_2_^•−^ + 2H^+^ → SOD (Cu^2+^) + H_2_O_2_

SOD (Cu^2+^) + O_2_^•−^ → SOD (Cu^+^) + O_2_

The working principle of the biosensor is based on the amperometric signal linked to the first equation, generating the current that becomes more cathodic upon addition of O_2_^•−^, proportional to the change in concentration of superoxide radical in solution.

Chen and coworkers prepared a hemin-modified pyrolytic graphite electrode (hemin-PGE) and successfully applied it to the electrochemical determination of O_2_^•−^ [110]. This electrode was used to detect O_2_^•−^ produced by xanthine oxidase‒catalyzed hypoxanthine oxidation. Since the dismutation of superoxide generates H_2_O_2_, its possible interference was eliminated by catalase. The antioxidant activity of uric acid (i.e., emerging as the hypoxanthine oxidation product) was also detected using the hemin‒modified electrode, because uric acid was not oxidized directly on the electrode at the potential studied. 

The concerned electrode reactions were as follows:

Hemin (Fe^3+^) + O_2_^•−^ → Heme (Fe^2+^) + O_2_

Heme (Fe^2+^) → Hemin (Fe^3+^) + e^−^

The amperometric response of the electrode corresponded to the hemin-catalyzed oxidation of superoxide. The measurement of SOD activity or any other antioxidant activity was based on the detection of enhanced decomposition of superoxide.

**Figure 6:**
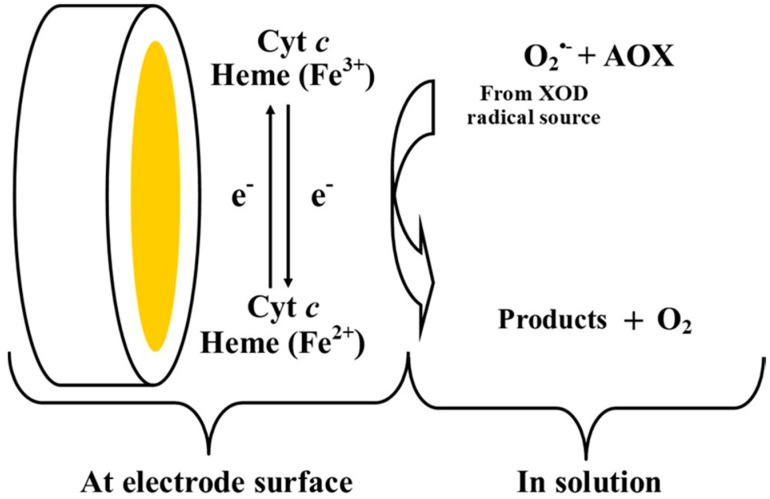
Cyt *c* sensor in determining superoxide scavenging activity of antioxidant (Cyt *c*: Cytchrome *c*; AOX: Antioxidant; HX: Hypoxanthine; XOD: Xanthine oxidase).

A cytochrome *c*-based electrochemical sensor was developed to indirectly determine antioxidant capacity by the measurement of superoxide concentration [111]. The mechanism of this method is the reduction of Cyt *c* immobilized on the surface of a multilayer electrode (i.e., consisting of alternating layers of Cyt *c* and poly(aniline-sulfonic acid) on a gold wire electrode) by O_2_^•−^ in solution. The electrochemical activity of Cyt *c* was claimed to increase with the number of layers on the electrode. Superoxide radical measurement involved the reaction between Cyt *c* and superoxide followed by further oxidation at the electrode surface. The oxidation current showed the changes in proportion to the O_2_^•−^ concentration in solution. The measurement of change of the superoxide concentration due to the addition of an antioxidant is shown in Figure 6.

Electrochemical biosensors applied to O_2_^•−^ detection are summarized in Table 5. 

### 2.3. Peroxyl Radical

Peroxyl radicals (ROO^•^) are mostly formed by a direct reaction of oxygen with alkyl radicals (R^•^) [122]. Autoxidations of a majority of biologically important compounds including lipids proceed by free-radical chain processes involving peroxyl radicals. ROO^•^ is soluble in membranes and is known to be the main generator of lipid peroxidation which causes diseases such as cancer and liver injury [123]. Nanoprobes or sensors developed for screening peroxyl radical scavenging ability of antioxidant compounds are very limited in literature. The possible reason for this scarcity may be the fact that most conventional peroxyl radical scavenging assays are based on the fluorescence decay of a suitable probe molecule (like fluorescein, the probe molecule of the “oxygen radical absorbance capacity” or ORAC assay of antioxidant activity measurement) upon ROO^•^ attack [124], and noble metal nanoparticles (like AuNPs) are known to quench fluorescence themselves [125]. A rare example in this regard (but not limited to ROO^•^ detection) was the portable ceria nano-particle based sensor (NanoCerac) assay for rapid and sensitive detection of food antioxidants [48]. The colorimetric determination of the scavenging activity of surface-adsorbed peroxyl and hydroperoxyl radicals was accomplished by quantifying the ability of antioxidants to inactivate these radicals from the ceria NPs surface. This indirect method could not quantify inhibition at low concentrations. It was generally not applicable to all antioxidants and was poorly reproducible. Identification of peroxyl radicals (ROO^•^) generated by the adsorption of O_2_ onto unmodified or polyethylene glycol (PEG)‒functionalized single-walled carbon nanotubes (SWCNTs) in a cell-free system was performed [126]. The SWCNT-mediated oxidation of 2’,7’-dichlorodihydrofluorescein (H_2_DCF) to 2’,7’-dichloro-fluorescein (DCF) was accurately quantified by capillary electrophoresis (CE). The high separation power of CE eliminated the fluorescence quenching exerted by SWCNTs—which poses a main problem to microplate readers—and enabled the screening of quite different ROS scavengers, including small molecules, surfactants and proteins.

In the presence of certain ROS scavengers, inhibition of H_2_DCF was monitored, where oxidation of H_2_DCF was significantly weakened by α-tocopherol, ascorbic acid and trolox. DMSO (hydroxyl radical suppressor) and sodium azide (singlet oxygen scavenger) did not show any effect on DCF production. This method provides enhanced detection specificity by CE system.

### 2.4. Hydroxyl Radical

#### 2.4.1. Spectroscopic Sensors and Nanoprobes

Among ROS, hydroxyl radical (^•^OH) is known as the most reactive short-lived species with the highest redox potential, which can oxidize many different biological molecules including amino acids, sugars, lipids, proteins, and DNA. Two basic strategies for ^•^OH detection involve the measurement of (i) probe conversion under radical attack, and (ii) generation of long-lived radical species with a scavenger followed by reaction with the probe [127]. Adding to the advantages of fluorescence probes in specificity and sensitivity, ratiometric nanoprobes are used to resolve certain shortcomings and limitations of available probes for monitoring and detection of ^•^OH [128,129].

As the stability of fluorescent probes is sensitive to instrumental artifacts and environmental factors (e.g., temperature and pH), ratiometric detection can be utilized to reduce such limitations [130]. This detection uses the ratio of two signals: the first signal is given by a reporting dye that responds to reactive species while the second signal is given by a reference dye corrected for instrumental and environmental artifacts. The reporting dye can be encapsulated in polymeric nanocomposites. The proportion of these two fluorescence intensities (i.e., of analyte and reference signals) is independent of the probe concentration, permitting a more reliable and quantitative determination [131]. The selectivity of ^•^OH sensors and nanoprobes can be regulated by redox potentials; the exceptionally high potential of ^•^OH is compatible with selective spectroscopic probes which can only be converted at high potentials (e.g., of hydroxyl radicals) such that other ROS remain unsuccessful in oxidative probe conversion. A good example of sensor selectivity toward hydroxyl radicals was given by Bekdeşer et al. who used a terephthalate probe and a cupric-neocuproine sensor (on Nafion) for the detection of hydroxyl radicals and its scavenging by amino acids, thiol and plasma antioxidants [132], because terephthalate had no significant reactivity against other ROS such as superoxide anion radical, singlet oxygen and H_2_O_2_. In addition, the hydroxylation of terephthalate gave rise to a single hydroxylated product (i.e., 2-hydroxyterephthalate) instead of a mixture of isomers, due to the symmetrical structure of the probe. Immobilized Cu(II)-neocuproine complex on a Nafion membrane [5] was reduced to Cu(I)-Nc chelate as a result of reaction with the hydroxylated probe. Scavengers competed with terephthalate for ^•^OH and attenuated probe hydroxylation giving rise to a drop in sensor absorbance, indicative of ^•^OH scavenging activity of antioxidants. The analytical performances of ^•^OH nanoprobes and sensors are comparatively listed in Table 6.

King and Kopelman developed a ratiometric nanoprobe for detecting ^•^OH, using coumarin-3-carboxylic acid (C3C) as a reporter dye capable of direct reaction with ^•^OH to generate the highly fluorescent product 7-hydroxy-C3C. For ratiometric measurements, Texas Red-dextran (reference dye) was encapsulated within amine-functionalized polyacrylamide nanoparticles, where the reporter dye C3C was covalently attached to amine groups on nanoprobe [133]. In a similar study performed by Ganea et al., ratiometric COumarin-NEutral Red (CONER) nanoprobe was developed to detect ^•^OH in vitro using viable breast cancer cells [130]. Neutral red (reference dye) was encapsulated within biocompatible poly (lactide-co-glycolide) (PLGA) nanoparticles. Hydroxyl radical concentration was calculated as a function of the fluorescence intensity ratio (I_450_/I_528_) at the emmision wavelengths of 7-OH-C3C (450 nm) and neutral red (528 nm) when excited at λ_ex_ = 410 nm. Since ^•^OH could only react with C3C existing on the nanoparticle surface, the formation of 7-OH-C3C would be hindered when C3C was encapsulated in the polymer matrix. The CONER nanoprobe was more selective to ^•^OH over other ROS including H_2_O_2_, ^1^O_2_, O_2_^•−^ and OCl^−^. 

In another study, a ratiometric fluorescence biosensor utilizing gold nanoclusters (AuNC) was developed for monitoring hydroxyl radicals in live cells by Zhuang et al. [134]. AuNC protected by bovine serum albumin (BSA) was used as a reference fluorophore and the organic molecule 2-[6-(4′-hydroxy)phenoxy-3H-xanthen-3-on-9-yl]benzoic acid (HPF) was the specific recognition element for ^•^OH. Although HPF was nearly nonfluorescent, the fluorescence of HPF product after reaction with ^•^OH (i.e., dianionic fluorescein) gradually increased with the addition of ^•^OH. The developed fluorescent sensor showed a high selectivity for ^•^OH over other ROS and RNS (such as O_2_^•−^, ^1^O_2_, OCl^−^, alkylperoxyl radical, peroxynitrite, H_2_O_2_ and ^•^NO), metal ions, and other small biological molecules (such as amino acids and glucose), as well as high accuracy and sensitivity with a low LOD around 0.68 μmol·L^−1^. On the other hand, Gomes et al. [128] reported that the fluorescence intensity of HPF increased in the presence of peroxynitrite anion, and that HPF was classified as an advisable probe for determining ^•^OH, peroxynitrite anion and HOCl [137]. 

Li et al. developed a sandwich-structured upconversion nanoparticles (SWUCNPs) sensor for in vivo determination and monitoring of ^•^OH concentration [133]. These nanoparticles (doped with rare earth metal ions) had a successful performance in biosensing and bioimaging applications due to their deeper tissue diffusion and excitation within near-infrared range where biomolecules showed negligible auto-fluorescence. The mechanism of the nanoprobe was associated with NIR-light excited luminescence resonance energy transfer-based sensing. The nanoprobe was composed of two functionalities: upconversion NPs with sandwich structure and bared surface as the energy donor, and mOG, a modified Orange G azo dye with tunable light absorption, serving as both the energy acceptor and the ^•^OH recognizing ligand. The energy transfer from SWUCNPs to mOG was inhibited because of the oxidation and degradation of azo dye in the presence of ^•^OH. The sandwich structure of the nanoprobe significantly enhanced the extent of luminescence quenching, and consequently, extremely low levels of hydroxyl radical (LOD ≈ 1.2 fmol·L^−1^) could be detected. The SWUCNPs were also claimed to exhibit high selectivity and stability, low cytotoxicity and favorable cellular uptake. 

Surface modification of quantum dots could provide an improvement to the sensitive and selective detection of ROS, especially ^•^OH. Adegoke and Nyokong synthesized different thiol-capped (thioglycolic acid, 3-mercaptopropionic acid, glutathione) luminescent CdTe and CdTe/ZnS QDs for ^•^OH detection. A decrease in the fluorescence of QDs was due to electron transfer from QDs to ^•^OH having a high electron affinity, and CdTe@ZnS had a better performance than CdTe QDs in this regard, because ZnS increased the non-radiative recombination pahtway providing an increment in quenching efficiency. GSH-CdTe@ZnS was found to be the most sensitive QDs with LOD = 85 nmol·L^−1^. The CdTe@ZnS QDs had limited selectivity of fluorescence quenching toward hydroxyl radicals over H_2_O_2_ and ONOO^−^ [135]. 

#### 2.4.2. Electrochemical Nanosensors

Wang and coworkers developed a simple and sensitive electrochemical method for the determination of antioxidant capacity *via* nanoparticles [138]. In this study, 4-hydroxybenzoic acid (4-HBA) was used as a trapping agent for photogenerated (i.e., produced by photocatalytic oxidation of water with TiO_2_-NPs) ^•^OH radicals, leading to 3,4-dihydroxybenzoic acid (3,4-DHBA) subsequently measured by square wave voltammetry (SWV). After addition of antioxidant compounds, the 4-HBA probe competed with antioxidants for ^•^OH radicals, and consequently, the peak current due to 3,4-DHBA was decreased, depending on the ^•^OH scavenging ability of antioxidants. The IC_50_ values (as 50% scavenging concentrations for hydroxyl radicals) of different antioxidant compounds were calculated for trolox, lipoic acid, caffeic acid, glutathione, ascorbic acid, purified mint flavonoids, crude mint flavonoids and dandelion phenolics as; 22.15 µmol·L^−1^, 1.75 µmol·L^−1^, 2.72 µmol·L^−1^, 13.52 µmol·L^−1^, 60.55 µmol·L^−1^, 1.0 mg L^−1^, 1.1 mg L^−1^ and 1.3 mg L^−1^, respectively. The developed electrochemical method provided a good alternative for antioxidant assessment due to its fast response, accuracy and inexpensive apparatus.

The production of ^•^OH at a palladium oxide nanoparticles-modified ITO electrode was investigated during concomitant reduction of palladium oxide and H_2_O_2_ [139]. The catalytic current was shown to stem from the reoxidation of freshly exposed palladium metal by hydroxyl radicals. In the presence of ^•^OH scavenging antioxidants, the catalytic reduction current decreased. As a result, the hydroxyl radical scavenging activity of antioxidants were sorted as; lipoic acid > glutathione > gallic acid > Vitamin E > Vitamin C > uric acid > Trolox, and the detection limits for these antioxidants were 0.5, 0.5, 10, 25, 25, 25, 25 µmol·L^−1^, respectively. Since palladium oxide reduction occurred at negative potentials where antioxidants are usually not oxidized, this electrochemical method proved to be more selective toward antioxidants than other similar methods exploiting electrochemical oxidation of antioxidants at anodic potentials. 

### 2.5. Reactive Nitrogen Species

Reactive nitrogen species (RNS) take part in cell signalling under various physiological conditions and also provide host defense against bacterial and fungal pathogens [140]. While neither ^•^NO nor O_2_^•−^ are strong oxidants, ONOO^−^ is highly reactive and can oxidize DNA, proteins and lipids [141]. Unfortunately, very few efforts have been spent to detect RNS by selective nanoprobes. One non-specific application makes use of reduced glutathione (GSH) on the surface of AuNPs that can readily be desorbed via formation of glutathione disulfide (GSSG) upon ROS/RNS attack, and destabilized particles can aggregate to generate the plasmonic couplings between AuNPs that trigger a bathochromic shift in the visible spectrum and solution color change to blue [140]. The absorbance difference in the absence and presence of NO^•^ (i.e., ΔA_525nm_) showed a linear dependence (R^2^ = 0.9873) on ROS concentration ranging between 1.29 µmol·L^−1^−1.29 mol·L^−1^, with a LOD of 129 µmol·L^−1^ (for NO^•^). For the reaction kinetics of the nanoprobe with ROS/RNS, the change in absorbance with time was examined for fixed reaction conditions. For the non-radical oxidant, hydrogen peroxide, the proposed method could be more efficiently applied by converting H_2_O_2_ into measurable ROS (basically ^•^OH) via Fenton reaction. In the test system, H_2_O_2_, ^•^OH, and ClO^‒^ proved to be stronger and faster oxidants than NO^•^ and O_2_^•‒^. Using this system, the authors claimed to better distinguish cancerous and normal cells, based on their differential ROS and RNS production. Pu et al. developed a semiconductor NP-based NIR probe for RNS, relying on the resistance of the reporter cyanine dye to degradation by RNS, but although the fluorescent nanoprobe was capable of real-time fingerprinting of RNS in the tissues of living mice, it was not specific for RNS [142]. The cyanine dye derivative was attached to the semiconductor nanoparticle surface by a carbodiimide-activated coupling reaction. Thus, the surface of the semiconducting core, which did not react with ROS/RNS under normal conditions, was coated with fluorophore molecules (with a primary emission peak at 678 nm), and in the presence of ROS/RNS species in the medium, the fluorophore groups were disrupted to increase the emission intensity (due to the abolishment of fluorescence resonance energy transfer (FRET) within the nanoprobe). As a result, with the exception of H_2_O_2_, an increase was found in the signals for ONOO^‒^, ClO^‒^, ^•^OH, O_2_ and NO^•^, and quite linear results (R^2^ > 0.99) were obtained up to 0.5 μmol·L^−1^ concentrations, with a LOD as low as 10 nmol·L^−1^. From the emission signals, it was observed that the antioxidant N-acetylcysteine effectively scavenged both ClO^‒^ and ONOO^‒^. Chen et al. [143] developed a colorimetric probe of gold nanoparticles for the detection of peroxynitrite and its scavengers, based on the stabilizing effect of ssDNA on AuNPs. Citrate-stabilized AuNPs were further stabilized by adsorption of ssDNA, exhibiting the normal LSPR band in red colored solution. As peroxynitrite (ONOO^‒^) attack on AuNPs broke up ssDNA, the color of the solution turned to blue, because the formation of smaller DNA fragments eliminated the stabilizing effect, causing aggregation of NPs at high ionic strength. The absorbance signals were linear with respect to peroxynitrite concentration (R^2^ = 0.998) between 9.8 and 20.8 mmol·L^−1^. Antioxidants showed a restoration effect by scavenging peroxynitrite in the order of ascorbic acid > gallic acid > caffeic acid, and turning the solution color to red. The ONOO^‒^ scavenging ability of ascorbic acid showed a linear concentration dependence between 3.2 and 12.8 μmol·L^−1^, with a determination coefficient of (R^2^ = 0.986). 

Electrochemical nanosensors were claimed to provide better selectivity for RNS over other reactive species. For example, Quinton et al. fabricated a gold ultramicroelectrode network (Au-UME) for the separate determination of ^•^NO and ONOO^‒^, where they electrodeposited thin bilayers of poly(eugenol) and poly(phenol) on UME network for electrochemical detection of ^•^NO at a potential of 0.8 V (less interfered by other similar substances) versus Ag/AgCl in PBS, while they used uncoated Au-electrode to detect ONOO^‒^ at −0.1 V versus Ag/AgCl in the same buffer [144]. It was known from literature that bilayer coatings of poly(eugenol) and poly(phenol) is selective for ^•^NO [145]. The modified electrodes (i.e., Pt ultramicroelectrodes modified with electropolymerized phenol, eugenol, *o*-phenylenediamine and aniline thin layers) gave a linear calibration (R^2^ = 0.997) with a LOD value of 27 nmol·L^−1^ at a signal-to-noise ratio of 3. Nitrite, hydrogen peroxide, ascorbic acid and dopamine did not interfere, while fibronectin caused 20% decrease in sensitivity. Cruz et al. described an electrochemical DNA sensor for measuring the antioxidant activity against nitric oxide (^•^NO) radicals. The sensor consisted of dA_20_ (ssDNA composed of 20 adenine moieties) adsorbed onto carbon paste electrode (CPE). When this dA_20_-CPE was damaged upon ^•^NO attack, the peak current at +1.20 V versus Ag/AgCl (due to undamaged DNA-based material) decreased, whereas antioxidants (i.e., ascorbic acid and phenolic-cinnamic acids) restored the signal by nitric oxide scavenging [46]. For increasing concentrations of ^•^NO ranging between 1.8 and 180 μmol·L^−1^, DNA damage increased from 21 to 79%. The protective effect of ascorbic acid within the concentration range 1–20 mg L^−1^ gave a linear calibration curve (R^2^ = 0.988) with a LOD of 0.23 mg L^−1^, and reproducibility (RSD) of 4.9% at 10 mg L^−1^ concentration. 

## 3. Conclusions and Future Trends

In this review, innovative spectroscopic and electrochemical sensors and nanoprobes for the characterization of food antioxidants have been described, with special emphasis on home-made colorimetric sensors of antioxidant capacity/activity assessment. Colorimetric CUPRAC, ferric-phenanthroline and DMPD sensors that we developed effectively measure antioxidant and oxidant species on a cation-exchanger membrane, respectively, as simple as a pH paper showing solution acidity. Our colorimetric silver nanoparticle probe estimates antioxidant capacity with an electron-transfer mechanism, while the DTNB-functionalized gold nanoparticles exchange thiols enabling their colorimetric sensing. This review also summarizes the measurement of antioxidant activity of ROS/RNS scavengers with the aid of spectroscopic and electrochemical nanoprobes and sensors, however in most sensing applications designed for reactive species, the role of antioxidants acting as their scavengers has largely been neglected. Some of the future challenges regarding the design of new sensors and nanoprobes for food antioxidants should address specificity, sensitivity, stability and surface inactivation, response time and extended use, repeatability/reproducibility, and lack of linkage between science, technology and commercialization. More and more engineered nanoparticles are expected to be developed for specific purposes, and future trends may involve increased integration of antioxidant sensors to human health issues and development of point-of-care devices.

## Figures and Tables

**Table 1:** The Trolox-equivalent antioxidant capacities (TEAC) of selected antioxidants using absorptimetric- and reflectometric-CUPRAC sensors and solution-based method.

Antioxidant Compound	TEAC Absorptimetric-CUPRAC Sensor	TEAC Reflectometric-CUPRAC Sensor	TEAC Solution-CUPRAC
Quercetin	4.11	3.79	4.38
Morin	1.92	2.38	1.88
Fisetin	3.10	2.70	3.90
Catechin	1.92	1.16	3.09
Kaempferol	1.23	0.91	1.58
Rosmarinic acid	3.83	2.86	5.30
Gallic acid	2.10	1.76	2.62
Naringenin	0.60	0.42	0.05
Ascorbic acid	0.71	0.42	0.96

**Table 2:** Comparison of performances of DNA electrochemical sensors for total antioxidant capacity.

Sample	Target Layer	Free Radical	Linear Range	LOD	Advantages/Disadvantages	Ref.
Flavoured water containing ascorbic acid	Adenine Guanine	Hydroxyl radical	2–18 mg L^−1^ 0.5–5 mg L^−1^	0.1 mg L^−1^ 0.08 mg L^−1^	Advantage: -Reproducibility, constant sensitivity and avoidance of sample contamination, rapid and inexpensive evaluation of antioxidant capacity	[39]
Ascorbic acid	dsDNA	Hydroxyl radical	1.5–2.5 mmol·L^−1^	0.82 mmol·L^−1^	Disadvantage: -Narrow concentration range	[40]
Beverages	dA_21_	Hydroxyl radical	0.05–1.00 µmol·L^−1^	50 nmol·L^−1^	Advantage: -Easy, rapid, reproducible preparation, low detectability, TAC evaluation	[31]
Fruit flavoured water beverage	Guanine Adenine	Superoxide radical	1–5 mg L^−1^ 0.5–4 mg L^−1^	0.77 mg L^−1^ 0.5 mg L^−1^	Advantage: -Shorter detection time, smaller sample volume, higher accuracy, high simplicity and can be use without pretreatment	[35]
Fruit flavoured water beverage	Guanine Adenine	Sulfate radical	0.5–4 mg L^−1^ 0.5–4 mg L^−1^	0.47 mg L^−1^ 0.5 mg L^−1^	Advantage: Ease of preparation, rapid, reproducible. Disadvantage: Disposable	[45]
Orange-based beverages containing ascorbic acid	dA_20_-CPE	Nitric oxide radical	1–20 mg L^−1^	0.23 mg L^−1^	Advantage: -Inexpensive simple, stable and short response time	[46]
Pharm. antioxidant excipient in drugs (Na_2_S_2_O_5_)	Guanine	Hydroxyl radical	1–30 mmol·L^−1^	0.54 mmol·L^−1^	Advantage: -The surface area of Guanine/TiO_2_ NPs/MWCNTs/GCE has a large surface area and good electrochemical properties	[47]

**Table 3:** Comparison of the electroanalytical performance of different H_2_O_2_ nanosensors.

Electrode	Linear Range	LOD	Stability	Ref.
MWCNT/Ag nanohybrids/Au	0.05–17 mmol·L^−1^	50 µmol·L^−1^	90% remains after 30 days	[69]
AgNPs/DNA/GCE	4 µmol·L^−1^−16 mmol·L^−1^	1.7 µmol·L^−1^	30 days	[70]
Ag-DNA/GCE	2 µmol·L^−1^–2.5 mmol·L^−1^	0.6 µmol·L^−1^	Not reported	[72]
Ag-3D catalyst/G	50 µmol·L^−1^–2.5 mmol·L^−1^	1 µmol·L^−1^	92% remains after 30 days	[73]
AgNPs/CILE	2 µmol·L^−1^–250 µmol·L^−1^	0.7 µmol·L^−1^	Not reported	[74]
AgNPs/ZnONRs/FTO	8 µmol·L^−1^–1 mmol·L^−1^	0.9 µmol·L^−1^	Not reported	[75]
HRP/Ag/DNA/Au	1.5 µmol·L^−1^–2 mmol·L^−1^	0.5 µmol·L^−1^	15 days	[76]
AgNPs/GCE	0.01–0.9 mmol·L^−1^	2 µmol·L^−1^	Not reported	[77]
Hb/CIN-Chitosan/GCE	3.1 µmol·L^−1^–4 mmol·L^−1^	1.2 µmol·L^−1^	Not reported	[78]
Hb/ZrO_2_/DMSO/PG	1.5–30.2 µmol·L^−1^	0.14 µmol·L^−1^	Not reported	[79]
CuO-NPs/CILE	1 µmol·L^−1^–2.5 mmol·L^−1^	0.5 µmol·L^−1^	100 days	[80]
Nano-CuO/Nf/Pt	0.15 µmol·L^−1^–9 mmol·L^−1^	0.06 µmol·L^−1^	21 days	[81]
Pt_48_Pd_52_-Fe_3_O_4_ NPs/GCE	20 nmol·L^−1^–100 nmol·L^−1^	5 nmol·L^−1^	Not reported	[82]
Co_3_O_4_-NPs/GCE	4–80 nmol·L^−1^	0.4 nmol·L^−1^	20 days	[71]
PtNPs/PANI/MSF/ITO	1 µmol·L^−1^–2 mmol·L^-^	0.24 µmol·L^−1^	Not reported	[83]
Cu_2_O/Nf/Au	0.25 µmol·L^−1^–5 mmol·L^−1^	0.12 µmol·L^−1^	94.6% remains after 30 days	[84]
RGO-PANI-PtNPs/GCE	20 µmol·L^−1^–8 mmol·L^−1^	1.1 µmol·L^−1^	88% remains after 30 days	[85]
RGO/CuFe_2_O_4_/CPE	2–200 µmol·L^−1^	0.52 µmol·L^−1^	95.8% remains after 14 days	[86]
RGO/CeO_2_-AgNPs/SPE	0.5 µmol·L^−1^–12 mmol·L^−1^	0.21 µmol·L^−1^	90.7% remains after 42 days	[87]
Ag-MnO_2_-MWCNTs/GCE	5 µmol·L^−1^–10.4 mmol·L^−1^	1.7 µmol·L^−1^	90% remains after 30 days	[88]
PDDA/ERGO-ATP-PdNPs/GCE	0.1 µmol·L^−1^–10 mmol·L^−1^	0.016 µmol·L^−1^	Not reported	[89]
GN-CS/AuNPs/GCE	5 µmol·L^−1^–35 mmol·L^−1^	1.6 µmol·L^−1^	98.5% remains after 14 days	[90]

Abbreviations: MWCNT: Multiwalled carbon nanotubes; GCE: glassy carbon electrode; CILE: carbon ionic liquid electrode; ZnONRs: zinc oxide nanorods; FTO: fluorine-doped tin oxide; HRP: horseradish peroxidase; Hb: haemoglobin; CIN: carbon-coated iron nanoparticles; Nf: Nafion; DMSO: dimethyl sulfoxide; PG: pyrolytic graphite. MSF: mesoporous silica film; CPE: carbon-paste electrode; RGO: reduced graphene oxide; ERGO: electrochemically reduced graphene oxide; PtNP: platinum nanoparticles; SPE: screen-printed electrode; PDDA: Poly diallyldimethylammoniu chloride; Graphene: GN; Chitosan: CS.

**Table 4:** Nanoprobes and sensors for detecting superoxide anion radical.

Type of Material	Probe	Detection Type	LOD	Linear Range	Advantages/Disadvantages	Ref.
Carbon-dots	Hydroethidine	Fluorometric (λ_ex_ = 488 nm/ λ_em_ = 525 nm)	100 nmol·L^−1^	5 × 10^−7^–1.4 × 10^−4^ mol·L^−1^	Advantage: -Selective to O_2_^•−^ Disadvantage: -Could not be sensitive for quantification of O_2_^•−^	[92]
Ag@SiO_2_ core/shell nanoparticles	2-chloro-1,3-dibenzothiazoline cyclohexene (DBZTC)	Fluorometric	0.73 nmol·L^−1^	-	Advantage: -Sensitive detection towards other ROS (very high selectivity over hydrogen peroxide) -Applicable to living cells Disadvantage: Relatively low selectivity over NO^•^ and ONOO^‒^	[96]
Silica Sol-gel glass	Amplex Red-H_2_O_2_ (in SOD/HRP enzyme system)	Fluorometric (*λ*_exc_ = 530 nm/*λ*_em_ = 590 nm)	20 nmol·L^−1^	≤1.0 × 10^−6^ mol·L^−1^ of xanthine	Advantage: -This biosensor has ready-to-use and reusable properties Disadvantage: -The sensor is not expected to be of use in biological media where the concentration of hydrogen peroxide is relatively higher than that of superoxide	[97]
Gold nanoparticles	Cytochrome *c*	Surface Enhanced Raman Scattering (SERS)	10 nmol·L^−1^	2.3 × 10^−8^–2.8 × 10^−6^ mol·L^−1^	Advantage: -Suitable for qualitative/quantitative analysis of O_2_^•−^ in biosystems -The other ROS do not interfere with the method Disadvantage: -SERS signals may show relatively high RSD values	[99]
CdSe/ZnS quantum-dots	Cytochrome *c*	Fluorometric	-	0.08–1.49 μmol·L^−1^	Advantage: -High selectivity and specifity in living cells -Low cytotoxicity Disadvantage: -Cytochrome *c* may be reduced by a variety of biological electron carriers other than superoxide	[101]
Suffocated polystyrene nanoparticles/terbium-guanine nanoscale coordination polymers (PS-SO_3_H@Tb/G NCPs)	Terbium(III)	Fluorometric	3.4 nmol·L^−1^	10.12 nmol·L^−1^–6.0 μmol·L^−1^	Advantage: -High selectivity	[104]

**Table 5:** Biosensors for electrochemical detection of superoxide anion radical.

Biocomponent	Immobilization	Working Electrode ^a^ (Technique)	Radical Source	Linear Range	LOD	RSD (%)	Response Time	Stability	Advantages/Disadvantages	Ref.
SOD	Chemical cross-linking	Pt (Amp.)	XA/XOD	20–2000 µmol·L^−1^	10 µmol·L^−1^	-	60 s	30 days	Advantage: -Sensitive, reusable, stable; -First time immobilization of SOD on gelatin	[112]
SOD	Physical adsorption	H_2_O_2_ (Amp.)	XA/XOD	20–2000 µmol·L^−1^	10 µmol·L^−1^	≤5	≤100 s	≥7 days	Advantage: -Short response time, long life and robust -Easily miniaturized and perform in situ analyses Disadvantage: -Possible long-term degradation of functional biocomponents	[105]
SOD	Electropolymerization	GCE (Amp.)	KO_2_	20–3000 µmol·L^−1^	1 µmol·L^−1^	1.8 (n = 3)	-	2 months	Advantage: -Fast, selective response to O_2_^•−^, high sensivity, low detection limit	[109]
Hemin	Physical Adsorption	PGE (Amp.)	HX/XOD	-	-	6.4 (n = 12)	10–20 s	<5 h	Advantage: -Easily prepared and regenerated. High surface concn. of active species, but high current density	[110]
SOD	Electrospinnin, Physical adsorption	ITO (Amp.)	XA/XOD	0.5–2.5 µmol·L^−1^	0.3 µmol·L^−1^	<5	4 s	5 days	Advantage: -Low detection limit, shorter response time, good stability, reproducibility	[108]
SOD, Cys	Self-assembly monolayer	CFME (Amp.)	XA/XOD	13–105 nmol·L^−1^	-	-	< 5 s	7 days	Advantage: -Good stability, high selectivity, sensitivity for in vivo monitoring of O_2_^•−^ in biological systems	[113]
SOD	Sol-gel encapsulation, Self-assembly	Au (Amp.)	XA/XOD	0.05–0.4 µmol·L^−1^	-	4.8 (n = 7)	-	60 days	Advantage: -Easily manufactured, has high sensitivity and long-term stability	[114]
SOD	Sol-gel encapsulation	Au (Amp.)	XA/XOD	0.2–1.6 µmol·L^−1^	0.1 µmol·L^−1^	3.2 (n = 6)	-	1 month	Advantage: -Easily manufactured; low reduction potential minimizes interferences; high sensitivity and long-term stability	[115]
Zn-SOD	Electrodeposition	ITO (Amp.)	KO_2_	0.12–250 µmol·L^−1^	0.1 µmol·L^−1^	-	4 s	7 days	Advantage: -Biocompatible and easily prepared	[116]
Non-enzymatic	Pt nanoparticles covalently bonded to thiolated MWCNTs	GCE (Amp.)	KO_2_	0.1–3000 µmol·L^−1^	0.1 µmol·L^−1^	3.2 (n = 7)	3 s	1 month	Advantage: -Sensitive (low LOD) and selective determination of O_2_^•−^ without enzyme; wide linear range, good storage stability	[117]
SOD	Ultrasonic electrodeposition	GCE (Amp.)	XA/XOD	5.6–2700 nmol·L^−1^	1.7 nmol·L^−1^	4.4 (n = 10)	<5 s	44 days	Advantage: -Wide linear range, fast amperometric response, low LOD (nM), high selectivity for O_2_^•−^ detection	[118]
Cyt *c*	Self-assembly monolayer	Polycrystalline gold (Amp.)	HX/XOD	0.4–1.2 nmol·L^−1^	500–600 nmol·L^−1^	-	20 s	-	Advantage: -Sensitivity for nM O_2_^•−^ and μM H_2_O_2_ Disadvantage: -Less selective than SOD-based sensor and heme protein is not specific for O_2_^•−^	[119]
Cyt *c*	Self-assembly monolayer	Au (Amp.)	HX/XOD	0.4–1.5 µmol·L^−1^	-	-	5–8 s	-	Advantage: -Sensitive, fast and stable for O_2_^•−^ detection	[111]
Cyt *c*	Electrodeposition	NPG films covered SPE (Amp.)	XA/XOD	5–61 nmol·L^−1^	3.7 nmol·L^−1^	-	-	-	Advantage: -High sensitivity in measuring biologically released O_2_^•−^ Disadvantage: -Electrode is not sensitive to tiny amounts of O_2_^•−^ released extracellularly	[120]
Non-enzymatic	Physical adsorption (SDS-MWCNTs); Electrodeposition (AgNPs)	GCE (Amp.)	KO_2_	0.669–268 µmol·L^−1^	0.0897 nmol·L^−1^	2.61 (n = 5)	60 s	-	Advantage: -Sensitive and selective determination of O_2_^•−^ without enzyme; wide linear range, low detection limit; reproducible and stable -Can detect O_2_^•−^ released from living cells. Antioxidants were tested	[121]

^a^ Working electrode or transducer, SOD: Superoxide dismutase; XOD: Xanthine oxidase; XA: Xanthine; HX: Hypoxanthine; Cyt *c*: Cytchrome c; Cys: Cysteine; NPG: Nanoporous gold; SDS: Sodium dodecyl sulfate; MWCNTs: Multiwalled carbon nanotube composite; AgNPs; Silver nanoparticles; PGE: Pyrolytic graphite electrode; ITO: Indium-doped tin oxide; ME: Micro electrode; GCE: Glassy carbon electrode; CFME: Carbon fiber microelectrodes; SPE: Screen-printed electrode; RSD: Relative standard deviation.

**Table 6:** Spectroscopic nanoprobes and sensors for detecting hydroxyl radical.

Type of Material	Probe	Detection Type	LOD	Advantages/Disadvantages	Ref.
Poly lactide-co-glycolide nanoparticles	Coumarin-3-carboxylic acid Neutral red (reference dye)	Fluorometric	-	Advantage: -Compared with H_2_O_2_, HOCl, ^1^O_2_, probe is more selective for ^•^OH	[130]
Amine-functionalized polyacrylamide nanoparticles	Coumarin-3-carboxylic acid Texas Red-Dextran (reference dye)	Fluorometric	-	-	[133]
Upconversion nanoparticle	Modified orange G	Fluorometric	1.2 fmol·L^−1^	Advantage: -Low LOD -High selectivity and stability -Low cytotoxicity -Fine cellular uptake	[103]
Gold nanoparticle protected by BSA	2-[6-(4′-hydroxy)phenoxy-3H-xanthen-3-on-9-yl] benzoic acid	Fluorometric	0.68 μmol·L^−1^	Advantage: -Relatively low selectivity over ONOO^−^ and HOCl	[134]
Thiol-capped CdTe and CdTe/ZnS quantum dots	GSH-CdTe@ZnS MPA-CdTe@ZnS TGACdTe MPA-CdTe	Fluorometric	8.5 × 10^-8^ mol·L^−1^ (for GSH-CdTe@ZnS)	Advantage: -Reasonable selectivity over H_2_O_2_ and ONOO^−^	[135]
XAD-7 methacrylate	Nitrophenol	Spectrophotometric	Linear range: 3.6 × 10^−6^–8.0 × 10^−2^ mol·L^−1^	Advantage: -Multiple end product formation between nitrophenol and ^•^OH	[136]
Nafion membrane	Terephthalate	Spectrophotometric	-	Advantage: -Sensitive and specific biomarker due to single product formation	[132]

## References

[1] Ojeda C.B., Rojas F.S. (2006). Recent development in optical chemical sensors coupling with flow injection analysis. Sensors.

[2] Mac Craith B.D., Mcdonagh C., O’Keeffe G., Butler T., O’Kelly B., Mcgilp J.F. (1994). Fibre Optic Chemical Sensors Based on Evanescent Wave Interactions in Sol-Gel-Derived Porous Coatings. J. Sol-Gel Sci. Technol..

[3] Wolfbeis O.S. (1991). Fiber Optic Chemical Sensors and Biosensors.

[4] Apak R., Güçlü K., Özyürek M., Karademir S.E. (2004). Novel total antioxidant capacity index for dietary polyphenols and vitamins C and E, using their cupric ion reducing capability in the presence of neocuproine: CUPRAC method. J. Agric. Food Chem..

[5] Bener M., Özyürek M., Güçlü K., Apak R. (2010). Development of a low-cost optical sensor for cupric reducing antioxidant capacity measurement of food extracts. Anal. Chem..

[6] Bener M., Özyürek M., Güçlü K., Apak R. (2013). Novel optical fiber reflectometric CUPRAC sensor for total antioxidant capacity measurement of food extracts and biological samples. J. Agric. Food Chem..

[7] Bener M., Apak R. (2017). Ferric-o-phenanthroline adsorbed on a Nafion membrane: A novel optical sensor for antioxidant capacity measurement of food extracts. Sens. Actuators B Chem..

[8] Çekiç S.D., Avan A.N., Uzunboy S., Apak R. (2015). A colourimetric sensor for the simultaneous determination of oxidative status and antioxidant activity on the same membrane: *N*,*N*-Dimethyl-*p*-phenylene diamine (DMPD) on Nafion. Anal. Chim. Acta.

[9] Gavrilenko N.A., Saranchina N.V., Gavrilenko M.A. (2014). Polymethacrylate Colorimetric Sensor for Evaluation of Total Antioxidant Capacity. Procedia Chem..

[10] Steinberg I.M., Milardovic S. (2007). Chromogenic radical based optical sensor membrane for screening of antioxidant activity. Talanta.

[11] Blois M.S. (1958). Antioxidant Determinations by the Use of a Stable Free Radical. Nature.

[12] Brandwilliams W., Cuvelier M.E., Berset C. (1995). Use of a Free-Radical Method to Evaluate Antioxidant Activity. LWT-Food Sci. Technol..

[13] Apak R., Çapanoğlu E., Arda A.Ü., Guthrie B., Beauchamp J., Buettner A., Lavine B.K. (2015). Nanotechnological Methods of Antioxidant Characterization. The Chemical Sensory Informatics of Food: Measurement, Analysis, Integration.

[14] Vilela D., Gonzalez M.C., Escarpa A. (2015). Nanoparticles as analytical tools for in-vitro antioxidant-capacity assessment and beyond. Trac Trends Anal. Chem..

[15] Li Z.P., Duan X.R., Liu C.H., Du B.A. (2006). Selective determination of cysteine by resonance light scattering technique based on self-assembly of gold nanoparticles. Anal. Biochem..

[16] Li L., Li B. (2009). Sensitive and selective detection of cysteine using gold nanoparticles as colorimetric probes. Analyst.

[17] Üzer A., Durmazel S., Erçağ E., Apak R. (2017). Determination of hydrogen peroxide and triacetone triperoxide (TATP) with a silver nanoparticles—Based turn-on colorimetric sensor. Sens. Actuators B Chem..

[18] Vasileva P., Donkova B., Karadjova I., Dushkin C. (2011). Synthesis of starch-stabilized silver nanoparticles and their application as a surface plasmon resonance-based sensor of hydrogen peroxide. Colloid Surf. A Physicochem. Eng. Asp..

[19] Filippo E., Serra A., Manno D. (2009). Poly(vinyl alcohol) capped silver nanoparticles as localized surface plasmon resonance-based hydrogen peroxide sensor. Sens. Actuators B Chem..

[20] Endo T., Yanagida Y., Hatsuzawa T. (2008). Quantitative determination of hydrogen peroxide using polymer coated Ag nanoparticles. Measurement.

[21] Zhang Y., Li B., Xu C. (2010). Visual detection of ascorbic acid via alkyne-azide click reaction using gold nanoparticles as a colorimetric probe. Analyst.

[22] Özyürek M., Güngör N., Baki S., Güçlü K., Apak R. (2012). Development of a Silver Nanoparticle-Based Method for the Antioxidant Capacity Measurement of Polyphenols. Anal. Chem..

[23] Jiang Z.-J., Liu C.-Y. (2003). Seed-Mediated Growth Technique for the Preparation of a Silver Nanoshell on a Silica Sphere. J. Phys. Chem. B.

[24] Apak R., Çekiç S.D., Çetinkaya A., Filik H., Hayvalı M., Kılıç E. (2012). Selective Determination of Catechin among Phenolic Antioxidants with the Use of a Novel Optical Fiber Reflectance Sensor Based on Indophenol Dye Formation on Nano-sized TiO_2_. J. Agric. Food Chem..

[25] Güçlü K., Özyürek M., Güngör N., Baki S., Apak R. (2013). Selective optical sensing of biothiols with Ellman’s reagent: 5,5’-Dithio-bis(2-nitrobenzoic acid)-modified gold nanoparticles. Anal. Chim. Acta.

[26] Frens G. (1973). Controlled Nucleation for the Regulation of the Particle Size in Monodisperse Gold Suspensions. Nat. Phys. Sci..

[27] Üzer A., Can Z., Akın İ., Erçağ E., Apak R. (2014). 4-Aminothiophenol Functionalized Gold Nanoparticle-Based Colorimetric Sensor for the Determination of Nitramine Energetic Materials. Anal. Chem..

[28] Choleva T.G., Kappi F.A., Giokas D.L., Vlessidis A.G. (2015). Paper-based assay of antioxidant activity using analyte-mediated on-paper nucleation of gold nanoparticles as colorimetric probes. Anal. Chim. Acta.

[29] Barroso M.F., de-los-Santos-Alvarez N., Delerue-Matos C., Oliveira M.B.P.P. (2011). Towards a reliable technology for antioxidant capacity and oxidative damage evaluation: Electrochemical (bio)sensors. Biosens. Bioelectron..

[30] Prieto-Simon B., Cortina M., Campas M., Calas-Blanchard C. (2008). Electrochemical biosensors as a tool for antioxidant capacity assessment. Sens. Actuators B Chem..

[31] Barroso M.F., de-los-Santos-Alvarez N., Lobo-Castanon M.J., Miranda-Ordieres A.J., Delerue-Matos C., Oliveira M.B.P.P., Tunon-Blanco P. (2011). DNA-based biosensor for the electrocatalytic determination of antioxidant capacity in beverages. Biosens. Bioelectron..

[32] Barroso M.F., Delerue-Matos C., Oliveira M.B.P.P. (2011). Electrochemical DNA-sensor for evaluation of total antioxidant capacity of flavours and flavoured waters using superoxide radical damage. Biosens. Bioelectron..

[33] Mascini M., Palchetti I., Marrazza G. (2001). DNA electrochemical biosensors. Fresenius J. Anal. Chem..

[34] Mello L.D., Hernandez S., Marrazza G., Mascini M., Kubota L.T. (2006). Investigations of the antioxidant properties of plant extracts using a DNA-electrochemical biosensor. Biosens. Bioelectron..

[35] Labuda J., Bučková M., Vaníčková M., Mattusch J., Wennrich R. (1999). Voltammetric Detection of the DNA Interaction with Copper Complex Compounds and Damage to DNA. Electroanalysis.

[36] Korbut O., Bučková M., Tarapčík P., Labuda J., Gründler P. (2001). Damage to DNA indicated by an electrically heated DNA-modified carbon paste electrode. J. Electroanal. Chem..

[37] Bučková M., Labuda J., Šandula J., Križková L.V., Štěpánek I., Ďuračková Z. (2002). Detection of damage to DNA and antioxidative activity of yeast polysaccharides at the DNA-modified screen-printed electrode. Talanta.

[38] Qian P., Ai S., Yin H., Li J. (2010). Evaluation of DNA damage and antioxidant capacity of sericin by a DNA electrochemical biosensor based on dendrimer-encapsulated Au-Pd/chitosan composite. Microchim. Acta.

[39] Kamel A.H., Moreira F.T.C., Delerue-Matos C., Sales M.G.F. (2008). Electrochemical determination of antioxidant capacities in flavored waters by guanine and adenine biosensors. Biosens. Bioelectron..

[40] Zhang J.-J., Wang B., Li Y.-F., Jia W.-L., Cui H., Wang H.-S. (2008). Electrochemical Study on DNA Damage Based on the Direct Oxidation of 8-Hydroxydeoxyguanosine at an Electrochemically Modified Glassy Carbon Electrode. Electroanalysis.

[41] Barroso M.F., Delerue-Matos C., Oliveira M.B.P.P. (2012). Electrochemical evaluation of total antioxidant capacity of beverages using a purine-biosensor. Food Chem..

[42] Wang Y.M., Xiong H.Y., Zhang X.H., Wang S.F. (2012). Electrochemical study of bovine serum albumin damage induced by Fenton reaction using tris (2,2’-bipyridyl) cobalt (III) perchlorate as the electroactive indicator. Electrochim. Acta.

[43] Bian C.L., Xiong H.Y., Zhang X.H., Wen W., Wang S.F. (2011). An electrochemical biosensor for analysis of Fenton-mediated oxidative damage to BSA using poly-o-phenylenediamine as electroactive probe. Biosens. Bioelectron..

[44] Feng L.J., Zhang X.H., Zhao D.M., Wang S.F. (2011). Electrochemical studies of bovine serum albumin immobilization onto the poly-o-phenylenediamine and carbon-coated nickel composite film and its interaction with papaverine. Sens. Actuators B Chem..

[45] Barroso M.F., Delerue-Matos C., Oliveira M.B.P.P. (2011). Evaluation of the total antioxidant capacity of flavored water and electrochemical purine damage by sulfate radicals using a purine-based sensor. Electrochim. Acta.

[46] Cruz D., Barroso M.F., Ramalhosa M.J., Coelho A., da Silva H., Duarrte A.J., González-García M.B., Carvalho A.P., Delerue-Matos C. (2016). DNA-based sensor against nitrite oxide radical: Evaluation of total antioxidant capacity in beverages. J. Electroanal. Chem..

[47] Yue Y., Zhihong B., Sanming L., Kun Z. (2016). Electrochemical evaluation of antioxidant capacity in pharmaceutical antioxidant excipient of drugs on guanine-based modified electrode. J. Electroanal. Chem..

[48] Sharpe E., Frasco T., Andreescu D., Andreescu S. (2013). Portable ceria nanoparticle-based assay for rapid detection of food antioxidants (NanoCerac). Analyst.

[49] Ornatska M., Sharpe E., Andreescu D., Andreescu S. (2011). Paper Bioassay Based on Ceria Nanoparticles as Colorimetric Probes. Anal. Chem..

[50] Palaroan W.S., Bergantin J., Sevilla F. (2000). Optical fiber chemiluminescence biosensor for antioxidants based on an immobilized luminol/hematin reagent phase. Anal. Lett..

[51] Qiu H.M., Luo C.N., Sun M., Lu F.G., Fan L.L., Li X.J. (2012). A novel chemiluminescence sensor for determination of quercetin based on molecularly imprinted polymeric microspheres. Food Chem..

[52] Li Y., Li W., Zhou H., Wang F., Chen Y., Wang Y., Yu C. (2015). A facile method for the sensing of antioxidants based on the redox transformation of polyaniline. Sens. Actuators B Chem..

[53] Xu Y.L., Niu X.Y., Zhang H.J., Xu L.F., Zhao S.G., Chen H.L., Chen X.G. (2015). Switch-on Fluorescence Sensing of Glutathione in Food Samples Based on a Graphitic Carbon Nitride Quantum Dot (g-CNQD)-Hg^2+^ Chemosensor. J. Agric. Food Chem..

[54] Akshath U.S., Shubha L.R., Bhatt P., Thakur M.S. (2014). Quantum dots as optical labels for ultrasensitive detection of polyphenols. Biosens. Bioelectron..

[55] Rodrigues D.M.C., Ribeiro D.S.M., Frigerio C., Rodrigues S.S.M., Santos J.L.M., Prior J.A.V. (2015). Antioxidant capacity automatic assay based on inline photogenerated radical species from L-glutathione-capped CdTe quantum dots. Talanta.

[56] Liu H.L., Fang G.Z., Deng Q.L., Wang S. (2015). A triple-dimensional sensing chip for discrimination of eight antioxidants based on quantum dots and graphene. Biosens. Bioelectron..

[57] Wang Y., Lu J., Tang L., Chang H., Li J. (2009). Graphene Oxide Amplified Electrogenerated Chemiluminescence of Quantum Dots and Its Selective Sensing for Glutathione from Thiol-Containing Compounds. Anal. Chem..

[58] Zhao Q., Xiao C.B., Tu Y.F. (2015). The electrochemiluminescence of luminol on titania nanotubes functionalised indium tin oxide glass for flow injection analysis. Talanta.

[59] Schaferling M., Grogel D.B.M., Schreml S. (2011). Luminescent probes for detection and imaging of hydrogen peroxide. Microchim. Acta.

[60] He W.W., Zhou Y.T., Warner W.G., Hu X.N., Wu X.C., Zheng Z., Boudreau M.D., Yin J.J. (2013). Intrinsic catalytic activity of Au nanoparticles with respect to hydrogen peroxide decomposition and superoxide scavenging. Biomaterials.

[61] Li H., Ma X.Y., Dong J., Qian W.P. (2009). Development of Methodology Based on the Formation Process of Gold Nanoshells for Detecting Hydrogen Peroxide Scavenging Activity. Anal. Chem..

[62] Ma X.Y., Li H., Dong J.A., Qian W.P. (2011). Determination of hydrogen peroxide scavenging activity of phenolic acids by employing gold nanoshells precursor composites as nanoprobes. Food Chem..

[63] Chen Q.F., Rao Y.Y., Ma X.Y., Dong J.A., Qian W.P. (2011). Raman spectroscopy for hydrogen peroxide scavenging activity assay using gold nanoshell precursor nanocomposites as SERS probes. Anal. Methods.

[64] He D., Jones A.M., Garg S., Pham A.N., Waite T.D. (2011). Silver Nanoparticle-Reactive Oxygen Species Interactions: Application of a Charging-Discharging Model. J. Phys. Chem..

[65] He D., Garg S., Waite T.D. (2012). H_2_O_2_-Mediated Oxidation of Zero-Valent Silver and Resultant Interactions among Silver Nanoparticles, Silver Ions, and Reactive Oxygen Species. Langmuir.

[66] Liu J.Y., Hurt R.H. (2010). Ion Release Kinetics and Particle Persistence in Aqueous Nano-Silver Colloids. Environ. Sci. Technol..

[67] Bhatia P., Yadav P., Gupta B.D. (2013). Surface plasmon resonance based fiber optic hydrogen peroxide sensor using polymer embedded nanoparticles. Sens. Actuators B Chem..

[68] Zhang Y., Zhang Y.J., Xia X.D., Hou X.Q., Feng C.T., Wang J.X., Deng L. (2013). A quantitative colorimetric assay of H_2_O_2_ and glucose using silver nanoparticles induced by H_2_O_2_ and UV. Chin. Chem. Lett..

[69] Zhao W., Wang H., Qin X., Wang X., Zhao Z., Miao Z., Chen L., Shan M., Fang Y., Chen Q. (2009). A novel nonenzymatic hydrogen peroxide sensor based on multi-wall carbon nanotube/silver nanoparticle nanohybrids modified gold electrode. Talanta.

[70] Cui K., Song Y.H., Yao Y., Huang Z.Z., Wang L. (2008). A novel hydrogen peroxide sensor based on Ag nanoparticles electrodeposited on DNA-networks modified glassy carbon electrode. Electrochem. Commun..

[71] Salimi A., Hallaj R., Soltanian S., Mamkhezri H. (2007). Nanomolar detection of hydrogen peroxide on glassy carbon electrode modified with electrodeposited cobalt oxide nanoparticles. Anal. Chim. Acta.

[72] Wu S., Zhao H.T., Ju H.X., Shi C.G., Zhao J.W. (2006). Electrodeposition of silver-DNA hybrid nanoparticles for electrochemical sensing of hydrogen peroxide and glucose. Electrochem. Commun..

[73] He X.S., Hu C.G., Liu H., Du G.J., Xi Y., Jiang Y.F. (2010). Building Ag nanoparticle 3D catalyst via Na_2_Ti_3_O_7_ nanowires for the detection of hydrogen peroxide. Sens. Actuators B Chem..

[74] Safavi A., Maleki N., Farjami E. (2009). Electrodeposited Silver Nanoparticles on Carbon Ionic Liquid Electrode for Electrocatalytic Sensing of Hydrogen Peroxide. Electroanalysis.

[75] Lin C.Y., Lai Y.H., Balamurugan A., Vittal R., Lin C.W., Ho K.C. (2010). Electrode modified with a composite film of ZnO nanorods and Ag nanoparticles as a sensor for hydrogen peroxide. Talanta.

[76] Wang F.C., Yuan R., Chai Y.Q., Tang D.P. (2007). Probing traces of hydrogen peroxide by use of a biosensor based on mediator-free DNA and horseradish peroxidase immobilized on silver nanoparticles. Anal. Bioanal. Chem..

[77] Welch C.M., Banks C.E., Simm A.O., Compton R.G. (2005). Silver nanoparticle assemblies supported on glassy-carbon electrodes for the electro-analytical detection of hydrogen peroxide. Anal. Bioanal. Chem..

[78] Zhang H.-L., Zou X.-Z., Lai G.-S., Han D.-Y., Wang F. (2007). Direct Electrochemistry of Hemoglobin Immobilized on Carbon-Coated Iron Nanoparticles for Amperometric Detection of Hydrogen Peroxide. Electroanalysis.

[79] Liu S.Q., Dai Z.H., Chen H.Y., Ju H.X. (2004). Immobilization of hemoglobin on zirconium dioxide nanoparticles for preparation of a novel hydrogen peroxide biosensor. Biosens. Bioelectron..

[80] Ping J.F., Ru S.P., Fan K., Wu J.A., Ying Y.B. (2010). Copper oxide nanoparticles and ionic liquid modified carbon electrode for the non-enzymatic electrochemical sensing of hydrogen peroxide. Microchim. Acta.

[81] Miao X.M., Yuan R., Chai Y.Q., Shi Y.T., Yuan Y.Y. (2008). Direct electrocatalytic reduction of hydrogen peroxide based on Nafion and copper oxide nanoparticles modified Pt electrode. J. Electroanal. Chem..

[82] Sun X.L., Guo S.J., Liu Y., Sun S.H. (2012). Dumbbell-like PtPd-Fe_3_O_4_ Nanoparticles for Enhanced Electrochemical Detection of H_2_O_2_. Nano Lett..

[83] Ding L., Su B. (2015). A non-enzymatic hydrogen peroxide sensor based on platinum nanoparticle-polyaniline nanocomposites hosted in mesoporous silica film. J. Electroanal. Chem..

[84] Yan Z., Zhao J., Qin L., Mu F., Wang P., Feng X. (2013). Non-enzymatic hydrogen peroxide sensor based on a gold electrode modified with granular cuprous oxide nanowires. Microchim. Acta.

[85] Du X., Chen Y., Dong W., Han B., Liu M., Chen Q., Zhou J. (2017). A nanocomposite-based electrochemical sensor for non-enzymatic detection of hydrogen peroxide. Oncotarget.

[86] Benvidi A., Nafar M.T., Jahanbani S., Tezerjani M.D., Rezaeinasab M., Dalimasab S. (2017). Developing an electrochemical sensor based on a carbon paste electrode modified with nano-composite of reduced graphene oxide and CuFe_2_O_4_ nanoparticles for determination of hydrogen peroxide. Mater. Sci. Eng..

[87] Yao Z., Yang X., Wu F., Wu W., Wu F. (2016). Synthesis of differently sized silver nanoparticles on a screen-printed electrode sensitized with a nanocomposites consisting of reduced graphene oxide and cerium(IV) oxide for nonenzymatic sensing of hydrogen peroxide. Microchim. Acta.

[88] Han Y., Zheng J., Dong S. (2013). A novel nonenzymatic hydrogen peroxide sensor based on Ag-MnO_2_-MWCNTs nanocomposites. Electrochim. Acta.

[89] You J.M., Kim D., Kim S.K., Kim M.S., Han H.S., Jeon S. (2013). Novel determination of hydrogen peroxide by electrochemically reduced graphene oxide grafted with aminothiophenol-Pd nanoparticles. Sens. Actuators B Chem..

[90] Jia N., Huang B., Chen L., Tan L., Yao S. (2014). A simple non-enzymatic hydrogen peroxide sensor using gold nanoparticles-graphene-chitosan modified electrode. Sens. Actuators B Chem..

[91] Kehrer J.P. (1993). Free-Radicals as Mediators of Tissue-Injury and Disease. Crit. Rev. Toxicol..

[92] Gao X., Ding C., Zhu A., Tian Y. (2014). Carbon-dot-based ratiometric fluorescent probe for imaging and biosensing of superoxide anion in live cells. Anal. Chem..

[93] Wang X., Cao L., Yang S.-T., Lu F., Meziani M.J., Tian L., Sun K.W., Bloodgood M.A., Sun Y.-P. (2010). Bandgap-Like Strong Fluorescence in Functionalized Carbon Nanoparticles. Angew. Chem..

[94] Yang S.-T., Wang X., Wang H., Lu F., Luo P.G., Cao L., Meziani M.J., Liu J.-H., Liu Y., Chen M. (2009). Carbon Dots as Nontoxic and High-Performance Fluorescence Imaging Agents. J. Phys. Chem..

[95] Benov L., Sztejnberg L., Fridovich I. (1998). Critical evaluation of the use of hydroethidine as a measure of superoxide anion radical. Free Radic. Biol. Med..

[96] Li N., Wang H., Xue M., Chang C.Y., Chen Z.Z., Zhuo L.H., Tang B. (2012). A highly selective and sensitive nanoprobe for detection and imaging of the superoxide anion radical in living cells. Chem. Commun..

[97] Pastor I., Esquembre R., Micol V., Mallavia R., Mateo C.R. (2004). A ready-to-use fluorimetric biosensor for superoxide radical using superoxide dismutase and peroxidase immobilized in sol-gel glasses. Anal. Biochem..

[98] Zhou M., Diwu Z., Panchuk-Voloshina N., Haugland R.P. (1997). A Stable Nonfluorescent Derivative of Resorufin for the Fluorometric Determination of Trace Hydrogen Peroxide: Applications in Detecting the Activity of Phagocyte NADPH Oxidase and Other Oxidases. Anal. Biochem..

[99] Qu L.L., Li D.W., Qin L.X., Mu J., Fossey J.S., Long Y.T. (2013). Selective and Sensitive Detection of Intracellular O_2_^•−^ Using Au NPs/Cytochrome c as SERS Nanosensors. Anal. Chem..

[100] Zhang L., Huang D., Kondo M., Fan E., Ji H., Kou Y., Ou B. (2009). Novel High-Throughput Assay for Antioxidant Capacity against Superoxide Anion. J. Agric. Food Chem..

[101] Li D.W., Qin L.X., Li Y., Nia R.P., Long Y.T., Chen H.Y. (2011). CdSe/ZnS quantum dot–Cytochrome *c* bioconjugates for selective intracellular O_2_^•−^ sensing. Chem. Commun..

[102] Lee H., Lee K., Kim I.-K., Park T.G. (2009). Fluorescent Gold Nanoprobe Sensitive to Intracellular Reactive Oxygen Species. Adv. Funct. Mater..

[103] Li Z., Liang T., Lv S.W., Zhuang Q.G., Liu Z.H. (2015). A Rationally Designed Upconversion Nanoprobe for in Vivo Detection of Hydroxyl Radical. J. Am. Chem. Soc..

[104] Song Y., Hao J., Hu D., Zeng M., Li P., Li H., Chen L., Tan H., Wang L. (2017). Ratiometric fluorescent detection of superoxide anion with polystyrene@nanoscale coordination polymers. Sens. Actuators B Chem..

[105] Campanella L., Favero G., Persi L., Tomassetti M. (2000). New biosensor for superoxide radical used to evidence molecules of biomedical and pharmaceutical interest having radical scavenging properties. J. Pharm. Biomed. Anal..

[106] Ohsaka T., Shintani Y., Matsumoto F., Okajima T., Tokuda K. (1995). Mediated Electron-Transfer of Polyethylene Oxide-Modified Superoxide-Dismutase by Methyl Viologen. Bioelectrochem. Bioenerg..

[107] Tian Y., Mao L., Okajima T., Ohsaka T. (2002). Superoxide dismutase-based third-generation biosensor for superoxide anion. Anal. Chem..

[108] Santhosh P., Manesh K.M., Lee S.H., Uthayakumar S., Gopalan A.I., Lee K.P. (2011). Sensitive electrochemical detection of superoxide anion using gold nanoparticles distributed poly(methyl methacrylate)-polyaniline core-shell electrospun composite electrode. Analyst.

[109] Braik M., Barsan M.M., Dridi C., Ben Ali M., Brett C.M.A. (2016). Highly sensitive amperometric enzyme biosensor for detection of superoxide based on conducting polymer/CNT modified electrodes and superoxide dismutase. Sens. Actuators B Chem..

[110] Chen J., Wollenberger U., Lisdat F., Ge B.X., Scheller F.W. (2000). Superoxide sensor based on hemin modified electrode. Sens. Actuators B Chem..

[111] Beissenhirtz M.K., Scheller F.W., Lisdat F. (2004). A superoxide sensor based on a multilayer cytochrome c electrode. Anal. Chem..

[112] Emregul E. (2005). Development of a new biosensor for superoxide radicals. Anal. Bioanal. Chem..

[113] Tian Y., Mao L., Okajima T., Ohsaka T. (2005). A carbon fiber microelectrode-based third-generation biosensor for superoxide anion. Biosens. Bioelectron..

[114] Di J., Peng S., Shen C., Gao Y., Tu Y. (2007). One-step method embedding superoxide dismutase and gold nanoparticles in silica sol-gel network in the presence of cysteine for construction of third-generation biosensor. Biosens. Bioelectron..

[115] Di J., Bi S., Zhang M. (2004). Third-generation superoxide anion sensor based on superoxide dismutase directly immobilized by sol-gel thin film on gold electrode. Biosens. Bioelectron..

[116] Deng Z.F., Rui Q., Yin X., Liu H.Q., Tian Y. (2008). In vivo detection of superoxide anion in bean sprout based on ZnO nanodisks with facilitated activity for direct electron transfer of superoxide dismutase. Anal. Chem..

[117] Kim S.K., Kim D., You J.M., Han H.S., Jeon S. (2012). Non-enzymatic superoxide anion radical sensor based on Pt nanoparticles covalently bonded to thiolated MWCNTs. Electrochim. Acta.

[118] Wang L., Wen W., Xiong H.Y., Zhang X.H., Gu H.S., Wang S.F. (2013). A novel amperometric biosensor for superoxide anion based on superoxide dismutase immobilized on gold nanoparticle-chitosan-ionic liquid biocomposite film. Anal. Chim. Acta.

[119] Krylov A.V., Sczech R., Lisdat F. (2007). Characterization of antioxidants using a fluidic chip in aqueous/organic media. Analyst.

[120] Sadeghian R.B., Ostrovidov S., Han J., Salehi S., Bahraminejad B., Bae H., Chen M., Khademhossein A. (2016). Online monitoring of superoxide anions released from skeletal muscle cells using an electrochemical biosensor based on thick-film nanoporous gold. Am. Chem. Soc. Sens..

[121] Liu X., Liu X., Wei H., Song G., Guo H., Lu X. (2017). Sensitive detection of superoxide anion released from living cells using silver nanoparticles and functionalized multiwalled carbon nanotube composite. Sens. Actuators B Chem..

[122] Harman D. (2001). Aging: Overview. Ann. N. Y. Acad. Sci..

[123] Frei B. (1995). Cardiovascular disease and nutrient antioxidants: Role of low-density lipoprotein oxidation. Crit. Rev. Food Sci. Nutr..

[124] Ou B., Hampsch-Woodill M., Prior R.L. (2001). Development and Validation of an Improved Oxygen Radical Absorbance Capacity Assay Using Fluorescein as the Fluorescent Probe. J. Agric. Food Chem..

[125] Dulkeith E., Ringler M., Klar T.A., Feldmann J., Muñoz Javier A., Parak W.J. (2005). Gold Nanoparticles Quench Fluorescence by Phase Induced Radiative Rate Suppression. Nano Lett..

[126] Ren L., Kim H.K., Zhong W. (2009). Capillary Electrophoresis-Assisted Identification of Peroxyl Radical Generated by Single-Walled Carbon Nanotubes in a Cell-Free System. Anal. Chem..

[127] Li B.B., Gutierrez P.L., Blough N.V. (1999). Trace determination of hydroxyl radical using fluorescence detection. Method Enzymol..

[128] Gomes A., Fernandes E., Lima J.L.F.C. (2005). Fluorescence probes used for detection of reactive oxygen species. J. Biochem. Biophys. Methods.

[129] Soh N. (2006). Recent advances in fluorescent probes for the detection of reactive oxygen species. Anal. Bioanal. Chem..

[130] Ganea G.M., Kolic P.E., El-Zahab B., Warner I.M. (2011). Ratiometric coumarin-neutral red (CONER) nanoprobe for detection of hydroxyl radicals. Anal. Chem..

[131] Winterbourn C.C. (2014). The challenges of using fluorescent probes to detect and quantify specific reactive oxygen species in living cells. Biochim. Biophys. Acta-Gen. Subj..

[132] Bekdeşer B., Özyürek M., Güçlü K., Apak R. (2012). Novel spectroscopic sensor for the hydroxyl radical scavenging activity measurement of biological samples. Talanta.

[133] King M., Kopelman R. (2003). Development of a hydroxyl radical ratiometric nanoprobe. Sens. Actuators B Chem..

[134] Zhuang M., Ding C.Q., Zhu A.W., Tian Y. (2014). Ratiometric Fluorescence Probe for Monitoring Hydroxyl Radical in Live Cells Based on Gold Nanoclusters. Anal. Chem..

[135] Adegoke O., Nyokong T. (2012). A Comparative Study on the Sensitive Detection of Hydroxyl Radical Using Thiol-capped CdTe and CdTe/ZnS Quantum Dots. J. Fluoresc..

[136] Naughton D.P., Grootveld M., Blake D.R., Guestrin H.R., Narayanaswamy R. (1993). An Optical Hydroxyl Radical Sensor. Biosens. Bioelectron..

[137] Setsukinai K., Urano Y., Kakinuma K., Majima H.J., Nagano T. (2003). Development of novel fluorescence probes that can reliably detect reactive oxygen species and distinguish specific species. J. Biol. Chem..

[138] Wang Y., Calas-Blanchard C., Cortina-Puig M., Baohong L., Marty J.-L. (2009). An Electrochemical Method for Sensitive Determination of Antioxidant Capacity. Electroanalysis.

[139] Liu J., Lagger G., Tacchini P., Girault H.H. (2008). Generation of OH radicals at palladium oxide nanoparticle modified electrodes, and scavenging by fluorescent probes and antioxidants. J. Electroanal. Chem..

[140] Kumar S., Rhim W.K., Lim D.K., Nam J.M. (2013). Glutathione Dimerization-Based Plasmonic Nanoswitch for Biodetection of Reactive Oxygen and Nitrogen Species. ACS Nano.

[141] Dowding J.M., Dosani T., Kumar A., Seal S., Self W.T. (2012). Cerium oxide nanoparticles scavenge nitric oxide radical ((NO)-N-center dot). Chem. Commun..

[142] Pu K.Y., Shuhendler A.J., Rao J.H. (2013). Semiconducting Polymer Nanoprobe for In Vivo Imaging of Reactive Oxygen and Nitrogen Species. Angew. Chem.-Int. Edit..

[143] Chen L.J., Wu N., Sun B., Su H.C., Ai S.Y. (2013). Colorimetric detection of peroxynitrite-induced DNA damage using gold nanoparticles, and on the scavenging effects of antioxidants. Microchim. Acta.

[144] Quinton D., Girard A., Kim L.T.T., Raimbault V., Griscom L., Razan F., Griveau S., Bedioui F. (2011). On-chip multi-electrochemical sensor array platform for simultaneous screening of nitric oxide and peroxynitrite. Lab Chip.

[145] Quinton D., Porras-Gutiérrez A.G., Gutiérrez-Granados S., Griveau S., Bedioui F. (2010). Hybrid Materials from Electropolymerized Thin Polymer Layer-Based Electrodes for the Elaboration of a New Generation of Electrochemical Sensors of NO in Solution. ECS Trans..

